# An open-access multi-site fMRI dataset for investigating conscious visual perception

**DOI:** 10.1038/s41597-026-07377-y

**Published:** 2026-05-07

**Authors:** Aya Khalaf, David Richter, Yamil Vidal, Urszula Gorska-Klimowska, Rony Hirschhorn, Diptyajit Das, Kyle Sinan Taylan Kahraman, Praveen Sripad, Fatemeh Taheriyan, Liad Mudrik, Michael Pitts, Hal Blumenfeld, Floris P. de Lange, Niccolò Bonacchi, Tanya Brown, Lucia Melloni

**Affiliations:** 1 Department of Neurology, Yale School of Medicine, New Haven, CT USA; 2 Donders Institute for Brain, Cognition and Behaviour, Radboud University Nijmegen, Nijmegen, the Netherlands; 3 Mind, Brain and Behavior Research Center (CIMCYC), University of Granada, Granada, Spain; 4 Department of Psychiatry, University of Wisconsin-Madison, Madison, WI 53719 USA; 5 Sagol School of Neuroscience, Tel Aviv University, Tel Aviv, Israel; 6 Neural Circuits, Consciousness and Cognition Research Group, Max Planck Institute for Empirical Aesthetics, Frankfurt am Main, 60322 Germany; 7 Program for Brain, Mind, and Consciousness, Canadian Institute for Advanced Research, Toronto, Ontario Canada; 8 School of Psychological Sciences, Tel Aviv University, Tel Aviv, Israel; 9 Psychology Department, Reed College, Portland, OR 97202 USA; 10 Departments of Neurology, Neuroscience and Neurosurgery, Yale University School of Medicine, New Haven, CT USA; 11 William James Center for Research (WJCR), ISPA - Instituto Universitário, Rua Jardim do Tabaco, 34, 1149-041 Lisbon, Portugal; 12 Department of Neurology, New York University Grossman School of Medicine, New York, NY 10016 USA

**Keywords:** Perception, Consciousness

## Abstract

We present a functional magnetic resonance (fMRI) dataset collected as part of an adversarial collaboration aimed at arbitrating between the Global Neuronal Workspace theory (GNWT) and the Integrated Information Theory (IIT) of consciousness. Participants (N = 118) were presented with suprathreshold visual stimuli belonging to four different categories (faces, objects, letters, false fonts) with three orientations (front, left, right view), and three durations (0.5, 1.0, 1.5 seconds). Participants were asked to identify infrequent targets that changed in each block, thereby rendering two categories task-relevant and two task-irrelevant. The simplicity of the experimental design and of the task given to the participants ensures that these data are broadly reusable. Besides testing predictions from other theories of consciousness, these data can be used to examine various aspects of visual processing. The anonymized data were converted to Brain Imaging Data Structure (BIDS), and can be easily accessed through a web platform or an API. The dataset contains quality reports, demographics, behavioral performance, and eye-tracking data. We also provide code for preprocessing and analyzing the data.

## Background & Summary

Since the late 50 s, with the crisis of behaviorism and the advent of the cognitive revolution, the scientific study of the mind has flourished. But some aspects of the mind are more amenable to the scientific method than others. When cognitive neuroscience took shape in the late 70 s and early 80 s, the emergent discipline was mostly focused on the study of memory^1^, attention^2^, language^3^ and perceptual processes^4^.

During the 50 s, 60 s and 70 s, work in neuropsychology demonstrated that patients (amnesics, split-brain and blindsight) could perform tasks in response to stimuli that they failed to consciously perceive (or remember)^5^. But the study of the neural basis of consciousness in healthy populations remained challenging. This changed during the 90 s and early 2000s, when theoretical and technical advancements^6–8^, as well as a cultural shift (consciousness gradually became less of a taboo topic), opened new doors for the empirical study of consciousness. As functional magnetic resonance (fMRI) became available as a technique to measure brain activity^9,10^, it allowed the study of the neural basis of consciousness in healthy human participants with unprecedented detail^11–14^.

Since the 90 s, the expanding field of consciousness research has produced several competing theories^15^. While this theoretical expansion has merit, it also makes it harder to converge on an agreed-upon account, especially since many of these theories make incompatible predictions. Unfortunately, as theories have been developed and gathered empirical support largely independently, arbitrating between theories became a more difficult task^16^.

Recently, the Cogitate consortium has taken a direct step to address this issue^17^. Cogitate is a large-scale, open science, adversarial collaboration^18–20^ that has brought together proponents of the Integrated Information Theory (IIT)^21^ and Global Neuronal Workspace theory (GNWT)^22^ with the goal of experimentally contrasting them. To this end, the consortium has run two experiments, using functional magnetic resonance (fMRI), magnetoencephalography (MEG) and intracranial electroencephalography (iEEG).

This paper concerns the fMRI data included in the analyses of the first experiment (N = 118), which focused on the neural correlates of the content and duration of visual experience, under different task conditions^17,23^. The fMRI, eye-tracking and behavioral data presented here were collected at two independent and theory-neutral research centers, to ensure generalization across research devices, participant populations and to minimize bias. This fMRI dataset can be used in conjunction with the MEG-EEG and iEEG datasets released by the Cogitate consortium, opening possibilities for integration across techniques, for example using time-resolved methods^24^.

During the experiment, participants were presented with one visual stimulus at a time, which belonged to one of four different categories (faces, objects, letters and false fonts), and could be presented in one of three possible orientations. Stimuli were displayed foveally for either 0.5, 1 or 1.5 seconds, and were easy to perceive consciously. Participants were requested to report the presence of occasional targets. The rationale behind this form of presentation is that it poses an especially strong test to theories of consciousness, as a failure to confirm their predictions could not be explained by weak signals evoked by barely seen stimuli^17^. Furthermore, such methodology enables evaluating for the necessary mechanisms of consciousness postulated by the theories under testing.

Though the Cogitate Consortium^17^ focused on IIT and GNWT, these two theories are far from being the only theories proposed. For example, Seth & Bayne^15^ list a selection of 22 different theories.

Therefore, one of the goals of sharing these data is to make it possible to test specific predictions from other candidate theories of consciousness. The simple task used, in which clearly visible stimuli with different degrees of task relevance were presented to participants, results in data that holds the potential to assess many different theories of consciousness.

Importantly though, the potential for reusability of these data goes beyond the field of consciousness research. For example, these data could be used to study how visual feature encoding along the ventral stream can change depending on the relevance of the features for the task at hand, similarly to the work done by Duan *et al*.^25^, which shows that task-relevant and task-irrelevant features reach different depths in the processing hierarchy. As the stimuli presented in our task had three different durations, these data could also be used in the field of time perception, where it has been shown that the human supplementary motor area might contain “chronomaps”, in the timescale of the stimuli used in our task^26^.

We believe that the simplicity of the task and of the experimental design makes these data broadly useful to study different aspects of visual perception. Therefore here we make the full datasets available, in line with the FAIR (Findable, Accessible, Interoperable, Re-usable) principles^27^.

## Methods

### Participants

Data were collected from 120 participants; however, two participants were excluded due to incorrect tissue segmentation reconstruction and incomplete brain masking. Therefore, data from 118 participants (mean age 23.4 years, SD 3.6, 75 females, 3 left-handed) are released. The shared data include structural and functional images, eye-tracking, behavioral data, and standard operating procedures (SOPs) for data collection. Imaging was performed at the Yale Magnetic Resonance Research Center (MRRC) in New Haven, Connecticut, USA and at the Donders Centre for Cognitive Neuroimaging (DCCN) at Radboud University Nijmegen in the Netherlands. All participants provided written informed consent prior to their involvement in the study, including the eventual open sharing of their anonymized data. The study procedures adhered to the principles outlined in the Declaration of Helsinki. The experiment and the data collection process were reviewed and approved by the independent institutional ethics committees at each site (the Commissie Mensgebonden Onderzoek Regio Arnhem-Nijmegen at the Centre for Cognitive Neuroimaging at Donders Institute (NL45659.091.14); the Human Research Protection Program Institutional Review Board at Yale School of Medicine (2000027591).

### Experiment design

Participants performed a visual task designed to investigate brain activity associated with consciously perceived stimuli. The experimental design employed a factorial structure in which the factors of task relevance, stimulus duration, stimulus category, and stimulus orientation were manipulated. This design aimed to test theoretical predictions about stimuli that are clearly consciously perceived, irrespective of task demands, and to examine the persistence of brain activity over varying durations of stimulus presentation. The stimuli encompassed four categories: faces, objects, letters, and false fonts, each with 20 different identities. They were presented in three different orientations (front, left, and right views) and durations (0.5, 1.0, and 1.5 seconds). On each block (described below), two stimuli identities were presented to the participants and designated as targets. Participants were required to respond by pressing a button upon their presentation, regardless of their orientation. These could be either a face and an object, or a letter and a false font. This designation of targets served as a manipulation of task relevance, as it turns other stimuli from the same categories of the targets into task-relevant non-targets, and stimuli belonging to other categories into task-irrelevant.

### Stimuli

Faces were generated using FaceGen Modeler 3.1 and manipulated to ensure an even representation of male and female faces, with diverse representation of hairstyle and ethnicity (e.g., Caucasian, Asian, African American) to facilitate face identification. Object stimuli were selected from the Object Databank^28^. Both faces and objects were gray-scaled and adjusted for equal luminance and size using the SHINE toolbox^29^. Stimulus orientation was manipulated such that half of the stimuli from each category were presented in side views (equally split between right and left) and the other half were presented in front views (viewing angles: 0°, 30°, and −30°). Letters and false fonts were created using MAXON CINEMA 4D Studio (RC - R20) 20.059 and rendered in grayscale. Each font set (real font, false font) was displayed at three distinct horizontal viewing angles (0°, 30°, and −30°), designed with specific parameters including an extrusion depth of 9.79% of character height, a camera distance of 5.65 times character height, and an angle of 18° above the center of the letter, using a simulated focal length of 135 mm (35 mm equiv.). All stimuli were calibrated to occupy a visual angle of 6° by 6° within the participant’s field of view.

### Experiment procedure

The experiment consisted of 8 runs (~7.5 minutes per run), each comprising 4 blocks. Each run began with either two blocks of the Face/Object task, followed by two blocks of the Letter/False-font task, or vice versa, with the order counterbalanced across runs. Within each run, the blocks were separated by 3 baseline periods of 12 seconds each. Each block included 17 to 19 trials, consisting of 16 non-target trials (4 from each category) and 1 to 3 target trials. Non-targets included 8 task-relevant trials (from the same category as the targets but not the targets themselves) and 8 task-irrelevant trials (from different categories). A total of 576 trials were presented throughout the experiment.

**Fig. 1:**
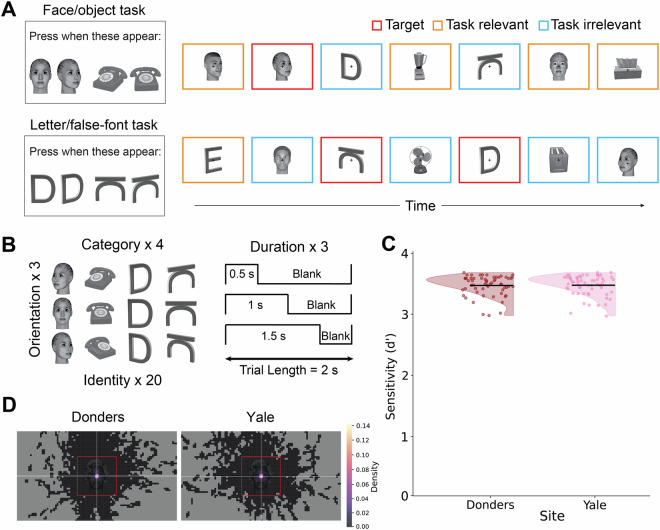
Experimental Design. (**A**) At the beginning of each block, a target screen displaying two targets side by side (a face and an object or a letter and a false font) was presented for 5 seconds. The participants were instructed to press a button whenever any of these targets appeared with any orientation in the subsequent sequence of trials. The presented trials within the sequence are categorized either as targets (highlighted in red), task-relevant stimuli (belonging to the same categories as the targets, depicted in orange), or task-irrelevant stimuli (belonging to the two other categories, depicted in light blue). The highlighted colors are for illustration purposes only and were not used in the paradigm presented to the participants. (**B**) Stimuli were manipulated across four dimensions including category (faces, objects, letters and false font), identity (each category contained 20 different stimuli), orientation (right, left, and front view), and duration (stimuli were presented for 0.5, 1.0, or 1.5 seconds). (**C**) The distribution of behavioral sensitivity scores (d’) is shown separately for each data acquisition site. Horizontal black lines indicate the average d’ for each site, while individual participant d’ scores are represented by dots. (**D**) Heatmaps of average fixation over the screen were generated over a 0.5-second window following stimulus onset, zoomed into the stimulus area for each recording site (Donders and Yale; overall accuracy < 2°: M = 86.45%, SD = 11.32).

Stimuli were displayed at the center of the screen for one of three durations: 0.5, 1, or 1.5 seconds. Following each stimulus, a blank screen was presented to extend trial duration to 2 seconds. Random jitter between trials was added (mean inter-trial interval of 3 seconds, jittered 2.5–10 seconds, with truncated exponential distribution). Participants were instructed to focus on a fixation cross displayed at the center of the screen during the entire duration of a run. Each block started with a target screen displaying two targets side by side, either a face and an object or a letter and a false font, depending on the task. The target screen was shown for 5 seconds, and participants were required to press a button whenever the targets appeared in the subsequent sequence of trials (see Fig. 1).

A standardized operating procedure (SOP) detailing the protocols for conducting the experiment was developed to ensure uniformity in data collection procedures across the two acquisition sites (Supplementary Information). The SOP encompassed detailed instructions for participant setup, equipment configuration, eye-tracking calibration, MR scanning sequences, as well as a step-by-step description of the experiment flow. Tailored SOPs were created for each site to accommodate specific variations in data acquisition procedures, while maintaining uniformity in shared protocols. These standardized approaches were developed to facilitate reliable comparison and utilization of data collected across different sites. Both experimental setups were extensively tested to ensure that presentation conditions are indeed standardized between sites, yielding comparable datasets^30^.

### Limitations

Caution is advised when performing analyses involving several factors (e.g. category × orientation × duration × task relevance), as this can result in a low number of trials per cell. The trial count per cell for one example participant can be found in Supplementary Table 1.

### Data acquisition

MRI data was acquired at both research centers on 3 T Prisma scanners (Siemens, Erlangen, Germany) using 32-channel head coils. Participants viewed the stimuli via an adjustable mirror mounted on the head-coil. At Yale, stimuli were presented using a Hyperion projection system (Psychology Software Tools; 1920 × 1080 pixels, 60 Hz; viewing distance ~113 cm) and an MRI-compatible BOLDscreen 32” IPS LCD monitor (Cambridge Research Systems; 1920 × 1080 pixels, 60 Hz; viewing distance ~134 cm) at the DCCN. To give their responses, participants used MRI-compatible button boxes, Current Design 2 × 2 (Yale) and Current Design 1 × 4 (DCCN). Stimuli were presented using Psychtoolbox v.3^31,32^ in Matlab R2019b (The Mathworks Inc., Natick, Massachusetts) on Windows 10 machines.

### Anatomical MRI data acquisition

Anatomical images were acquired using a T1-weighted magnetization prepared rapid gradient echo sequence (MP-RAGE): GRAPPA acceleration factor = 2, TR/TE = 2300/3.03 ms, voxel size 1 mm isotropic, FOV = 256 × 256 × 192 mm, 8° flip angle. More details on MR sequence parameters are shared in Supplementary Information.

### Functional MRI data acquisition

Functional images were acquired using a T2*-weighted EPI sequence: multiband factor = 4, TR/TE = 1500/39.6 ms, voxel size = 2.02 × 2.02 × 2 mm, FOV = 210 × 210 × 136 mm (68 slices), 75° flip angle, A/P phase encoding direction, BW = 2090 Hz/Px. Before each functional run a single band reference image was acquired. In addition, short sequences with inverted phase encoding direction were acquired while the participant was at rest at multiple points throughout the experiments.

### Eye tracking data acquisition

Gaze position and pupil size were recorded using an MRI-compatible EyeLink 1000 Plus eye-tracker (SR Research, Ottawa, Canada). Eye data was acquired at 1000 Hz, monocular, recording the left eye (DCCN) and right eye (Yale). A nine-point calibration was performed at the beginning of each experiment session and recalibrated as needed at the beginning of each block/run.

### Site-specific equipment differences

Efforts were made to minimize differences between the acquisition sites and standardize the setup as much as possible^30^. While the MRI scanners and eye-trackers used were of the same brand and model, the response boxes used were of the same brand, but had different button layout. Furthermore, the computers running the task, the display hardware and viewing distances differed slightly. Despite these minor discrepancies, participants managed to hold fixation correctly in both acquisition sites. Furthermore, we ran JZS Bayes Factor tests (*r = *0.707)^33^ to confirm that sensitivity index (BF_01_ = 5.07) and reaction times (BF_01_ = 3.27) did not differ across sites. In Figs. 4, 5, we report QC measures for each testing site independently, showing that the distributions are largely overlapping.

## Data Records

The data is provided in both raw and Brain Imaging Data Structure (BIDS^34^) formats and can be accessed through either 1) Archival Format: Zip Bundles or 2) “Live” Database Release: XNAT (eXtensible Neuroimaging Archive Toolkit^35^).

### Bundles

In the Bundles format, users can download prepared data packages and their metadata in a zip file, with options to access either the full dataset or a sample fMRI dataset.

#### Raw data structure

**Fig. 2:**
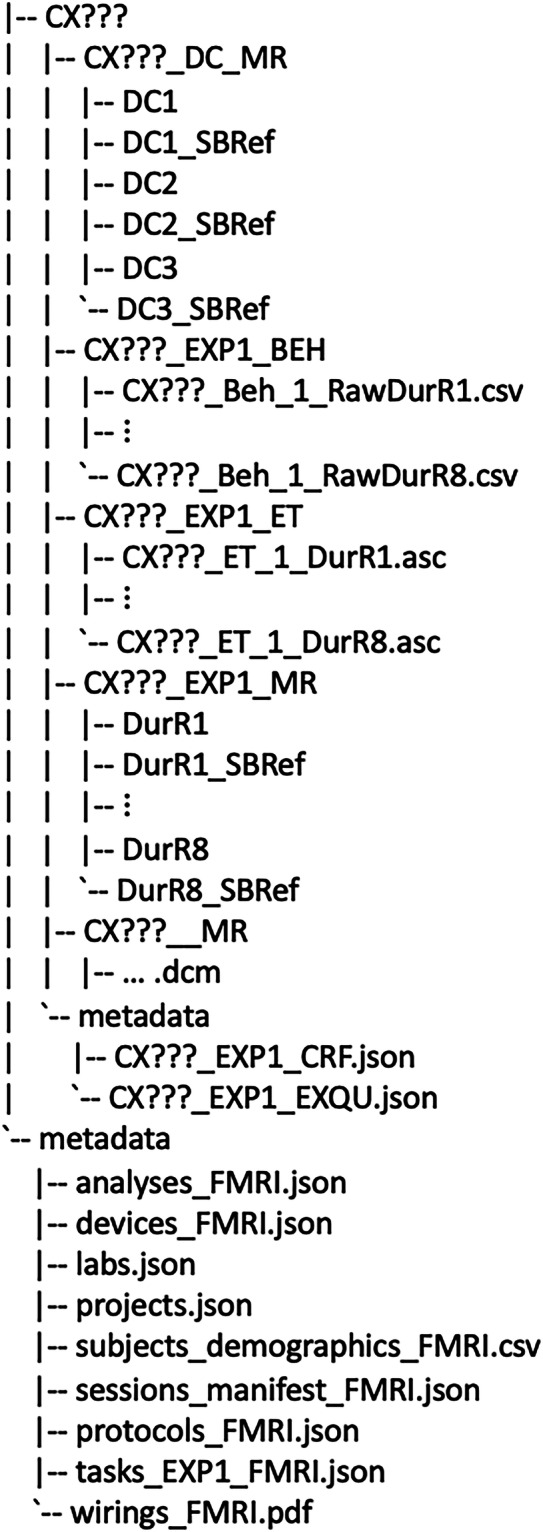
Structure of raw data bundles.

The organization of raw data bundles follows a specific naming convention and folder structure, which is detailed here. The project root is divided into sub-folders containing each participant’s data folder and a metadata folder, as illustrated in Fig. 2. Each participant’s folder is labeled with a unique ID in the format “CX???,” where ‘X’ indicates the collection site (‘C’ for DCCN and ‘D’ for Yale), and the question marks represent a 3-digit number, together forming the participant ID.

Each participant’s directory includes organized sub-directories containing individual-specific data, along with a metadata folder that holds documents such as the Case Report Form (CRF) and Exit Questionnaire (EXQU). The CRF was used to report any issues that occurred during the experiment, and the EXQU was completed by participants after the experiment to gather feedback on their experience related to the tasks performed. The data subfolders follow a structured naming pattern: SUBJECT_PARADIGM_MODALITY. Here, SUBJECT represents the participant ID, PARADIGM refers to the experimental paradigm, and MODALITY indicates the type of data contained within the folder. Anatomical MR scans are located in CX???__MR. As these data were collected independently of the task, they lack an experimental paradigm identifier; therefore, the PARADIGM field in the folder name is empty. Other folders include behavioral data (CX???_EXP1_BEH), eye-tracking data (CX???_EXP1_ET), and functional MR data (CX???_EXP1_MR) collected during Experiment 1. Additional scans were obtained for susceptibility distortion correction (CX???_DC_MR), and a single-band reference (SBRef) image was acquired before each scan, indicated by “SBRef” in the folder name.

The metadata folder in the root directory contains several key files, including the analysis code (analysis_FMRI.json), a list of data acquisition devices (devices_FMRI.json), labs.json file which includes metadata related to the project partner’s laboratories, such as the institutional name, location, contact information, and Principal Investigator details, projects.json file that contains metadata associated with the project, including the project start and end dates, funding details, project personnel, outputs, and ethical considerations, MR dataset manifest (sessions_manifest_FMRI.json), the experimental protocol (protocols_FMRI.json), participant demographic data (subjects_demographics_FMRI.json), descriptions of the experimental paradigm (tasks_EXP1_FMRI.json), and a wiring diagram for device connections (wirings_FMRI.pdf). Details on the raw data types and their formats are summarized in Table 1.

**Table 1:** Naming conventions and formats of raw data.

Data type	Folder	File naming conventions	Data formats
Behavioral data	CX???_EXP1_BEH	CX???_Beh_1_RawDurR?	CSV
Eye-tracking data	CX???_EXP1_ET	CX???_ET_1_DurR?	ASC
Anatomical MRI scans	CX???__MR	*.dcm	DICOM
Functional MRI scans	CX???_EXP1_MR	Dur?/*.dcm DurR?_SBRef/*.dcm	DICOM
Distortion Correction MRI Scans	CX???_DC_MR	DC?/*.dcm DC?_SBRef/*.dcm	DICOM

#### BIDS data structure

Raw data were formatted into BIDS using BIDSCoin^36^. The root directory contains subject-specific folders (e.g., sub-CX???) along with a derivatives folder and metadata files, including dataset_description.json, participants.json, participants.tsv, and README.md. The derivatives folder includes project-level metadata files such as analysis.json, devices_FMRI.json, FMRI_demographics.csv, FMRI_manifest.json, labs.json, projects.json, protocols.json, tasks_EXP1_FMRI.json, and wiring_FMRI.pdf. Subject-level metadata, including EXQU, CRF, and subject-specific demographics, are also located in the derivatives folder under each subject’s folder.

**Fig. 3:**
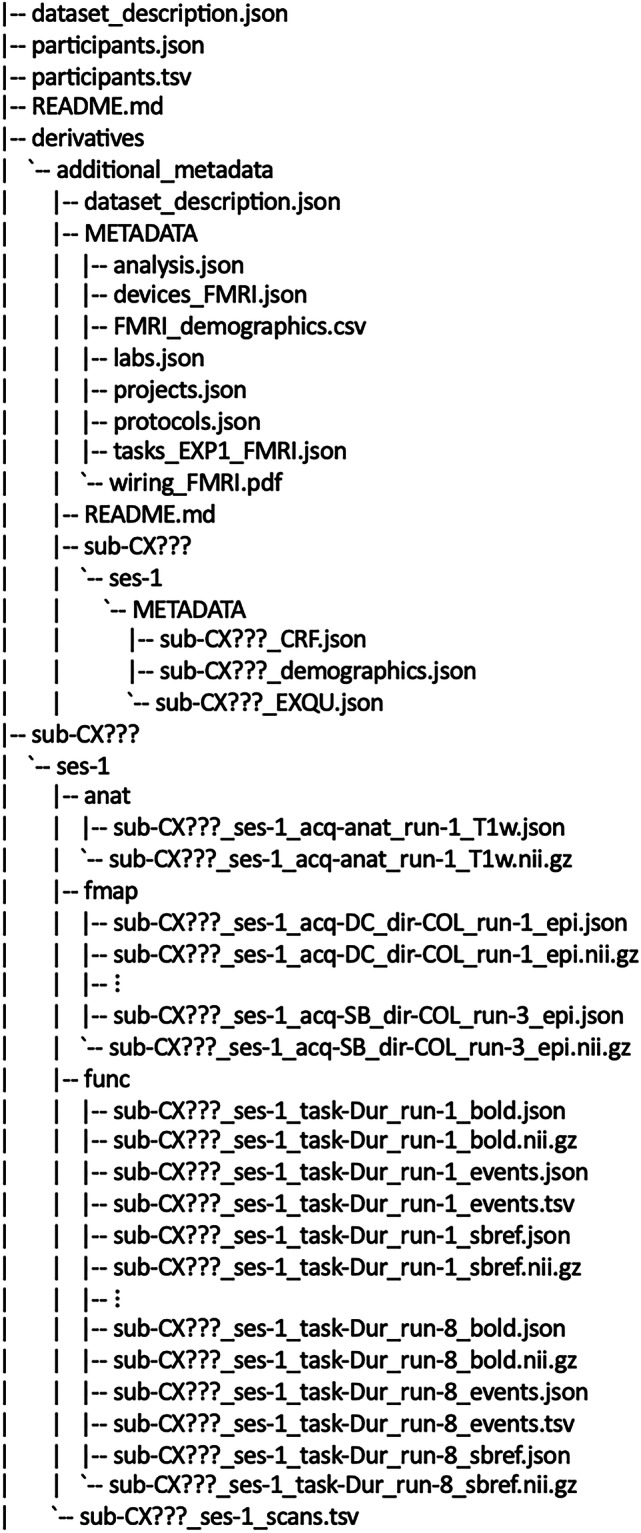
Structure of BIDS data bundles.

In the main directory, each subject folder contains a session subfolder (e.g., ses-1), which organizes data into categories: functional MRI (func), anatomical imaging (anat), and field maps (fmap) (see Fig. 3). The file types and formats of the data are detailed in Table 2.

**Table 2:** Naming conventions and formats of BIDS data.

Data type	Folder	File naming conventions	Data formats
Anatomical MRI data	anat	sub-CX???_ses-1_acq-anat_run-1_T1w	NIFTI and JSON
Fieldmap data	fmap	sub-CX???_ses-1_acq-DC_dir-COL_run-?_epi sub-CX???_ses-1_acq-SB_dir-COL_run-?_epi	NIFTI and JSON
Functional MRI data	func	sub-CX???_ses-1_task-Dur_run-?_bold sub-CX???_ses-1_task-Dur_run-?_sbref	NIFTI and JSON
Experimental events	func	sub-CX???_ses-1_task-Dur_run-?_events	TSV and JSON

Below is a breakdown of the files, with brief descriptions for each.

#### Root level

- **dataset_description.json:** General information about the BIDS version, dataset type, authors, acknowledgments, funding, ethics approvals, and the COGITATE website link.
- **participants.json:** Detailed participant information including biological sex, age, handedness, weight, height, primary and secondary languages, ethnicity, education level, colorblindness status, visual correction method, eye dominance, eye chart test results, and strength of visual correction in diopters.
- **participants.tsv:** A tab-separated values file containing similar metadata as participants.json.
- **README:** Overview of fMRI data, BIDS format, and dataset contents.

#### Derivatives directory

This directory contains additional metadata:

- **additional_metadata:** Contains all metadata.

- dataset_description.json: Details about BIDS version, dataset type and list of metadata in the additional_metadata folder.
- METADATA: Metadata files:

- analysis.json: Details analysis steps, their order and the analysis code repository link.
- devices_FMRI.json: Lists devices used for fMRI data acquisition.
- FMRI_demographics.csv: Demographic information for all participants
- FMRI_manifest.json: Comprehensive mapping of MRI scan labels categorized by subject and session
- labs.json: Metadata related to the project partner’s labs (e.g. Institutional name, location, contact information, and Principal Investigator details)
- projects.json: Metadata related to the project (e.g. project start/end date, funding details, project personnel, outputs, and ethics)
- protocols.json: Link to the COGITATE wiki.
- tasks_EXP1_FMRI.json: Information about the behavioral task, stimuli, and responses.
- wiring_FMRI.pdf: Wiring diagram of fMRI.

- README.md: Explanation of the additional_metadata directory.
- sub-CX???: participant-specific metadata:

- ses-1: The first session of data collection.
- METADATA: Further participant-specific metadata files:

- sub-CX???_CRF.json: Case report form.
- sub-CX???_demographics.json: Demographic information for the participant.
- sub-CX???_EXQU.json: Exit questionnaire form completed at the end of the experiment.

#### Subject-specific data

Each participant’s data is organized in their respective folder:

- **sub-CX???:** Data for the participant.

- ses-1: Indicates the first session of data collection.

- anat: Anatomical MRI images.
- sub-CX???_ses-1_acq-anat_run-1_T1w.json: Metadata for the anatomical image.
- sub-CX???_ses-1_acq-anat_run-1_T1w.nii.gz: NIfTI file containing the anatomical MRI image.
- fmap: Fieldmap images for distortion correction (DC).
- sub-CX???_ses-1_acq-DC_dir-COL_run-?_epi.json: Metadata for the fieldmap image.
- sub-CX???_ses-1_acq-DC_dir-COL_run-?_epi.nii.gz: NIfTI file with EPI images for distortion correction (DC).
- sub-CX???_ses-1_acq-SB_dir-COL_run-?_epi.nii.gz and sub-CX???_ses-1_acq-SB_dir-COL_run-?_epi.json: Single-band (SB) reference image associated with the inverted phase encoding direction scan (acq-DC) and its corresponding metadata
- func: Functional imaging data related to the task.
- sub-CX???_ses-1_task-Dur_run-?_bold.json: Metadata for the functional MRI BOLD time series data, including details on acquisition parameters.
- sub-CX???_ses-1_task-Dur_run-?_bold.nii.gz: NIfTI file containing the functional MRI BOLD time series.
- sub-CX???_ses-1_task-Dur_run-?_events.json and.tsv: Files detailing event onset times, durations, event types, task relevance, stimulus orientation and ID, and subject responses.
- sub-CX???_ses-1_task-Dur_run-?_sbref.json and.nii.gz: Single-band (SB) reference image and associated metadata (detailing acquisition parameters).
- sub-CX???_ses-1_scans.tsv: TSV file including time of acquisition.

### XNAT

This database provides a web interface for navigating data and an API (Application Programming Interface) for programmatically retrieving specific datasets based on user interests. XNAT^35^ offers flexibility, allowing researchers to download the entire dataset, specific modalities, or apply filters to extract and download subsets of data.

#### Raw data structure

In the project folder on XNAT, each participant’s data is organized under the “Subjects” table, identified by an ID in the format “CX???”. In the upper section of the project folder, under the “Details” tab, users can access additional metadata within the “Custom Fields” section.

Here is a breakdown of each section:

- **Lab:** Information on the labs involved in data acquisition, including principal investigators, institutions, departments, and contact details.
- **Project Ethics:** Details on the responsible member and institution, as well as the protocol number, approving committee, and start date.
- **Project:** Start and end dates for the project, along with the manager, team leader, and data curation team members, including the manager’s name.
- **Project Outputs:** Links to publications and other relevant resources associated with the project.
- **Project Funding:** Information on funding sources, including grants and supporting organizations.

Additional project-level metadata, such as analysis.json, devices_FMRI.json, FMRI_demographics.csv, FMRI_manifest.json, labs.json, projects.json, protocols.json, tasks_EXP1_FMRI.json, and wiring_FMRI.pdf, are available under the “Resources” tab.

Within each subject’s directory, users will find “EyeTracker” and “MR Session” in the “Experiments” table in the lower section. These contain eye-tracking data and MR scans, respectively, as their names suggest. Subject-level metadata, including demographic information, is available in a custom field for quick viewing and as a downloadable file under “Resources.”

In the “MR Sessions” section, users can access individual scans and download them. Additionally, CRF and EXQU data are provided as custom fields and are also available under “Resources,” along with behavioral data.

#### BIDS data structure

On XNAT, raw data is organized as follows: within the fMRI project, users will find a list of participants identified by IDs in the format sub-CX??? under the “Subjects” table along with additional information under the “Details” tab, similar to what was previously described in the Raw Data Structure section.

Each subject’s directory contains data organized similarly to the Bundles format. For data access, users can preview and download all files via “Resources” or “Manage Files.” For specific files or filtered downloads, users can select “Advanced”, located in the upper-right corner next to the search box.

#### Subject and experiment metadata

Subject and experimental metadata in XNAT are organized through custom forms accessible in the GUI. Subject-level metadata, which includes demographic information, is available under each subject’s page and can be downloaded from the “Resources” tab. Experiment-level metadata, including Case Report Forms (CRFs) and Exit Questionnaires (EXQUs), is accessible within each session and documents session quality and participant responses. Experiment-level metadata files are also downloadable from the session’s “Resources” tab.

## Technical Validation

### Quality control

DICOM data was converted to a BIDS compliant dataset using BIDScoin^36^, relying on dcm2niix (https://github.com/neurolabusc/dcm2niix). BIDS compliance was ensured using BIDS-Validator (https://github.com/bids-standard/bids-validator).

The data underwent a three-tier validation process, following the guidelines outlined in Gorska-Klimowska*, Hirschhorn* *et al*. (manuscript in preparation). First, we verified that all necessary files were present, adhered to consistent naming conventions, and were devoid of any personal identifiers. Second, we made sure each participant behaved as expected, by analyzing behavioral and eye-tracking data. The results indicated a high task performance, with an average hit rate of 97.50% (SD = 3.58) and a low incidence of false alarms (M = 0.66%, SD = 0.60) (Fig. 1C). Importantly, no participants fell below the predefined exclusion thresholds (hit rates < 70% or false alarm rates > 30%). Additionally, fixations remained relatively stable for all participants throughout the entire experiment (mean accuracy < 2° = 86.45%, SD = 11.32, Fig. 1D).

Data quality control was done with a combination of visual inspection of structural and functional images, quantification, and inspection of outputs of both MRIQC^37^, and fMRIPrep^38,39^. Two datasets with clear artifacts of the incorrect reconstruction of anatomical scans, substantial signal dropout and distortion were excluded from the shared sample. The rest of the data underwent a quality control assessment summarized below. All shared data were defaced, while the QC metrics were performed on non-defaced data.

### Anatomical scans

**Fig. 4:**
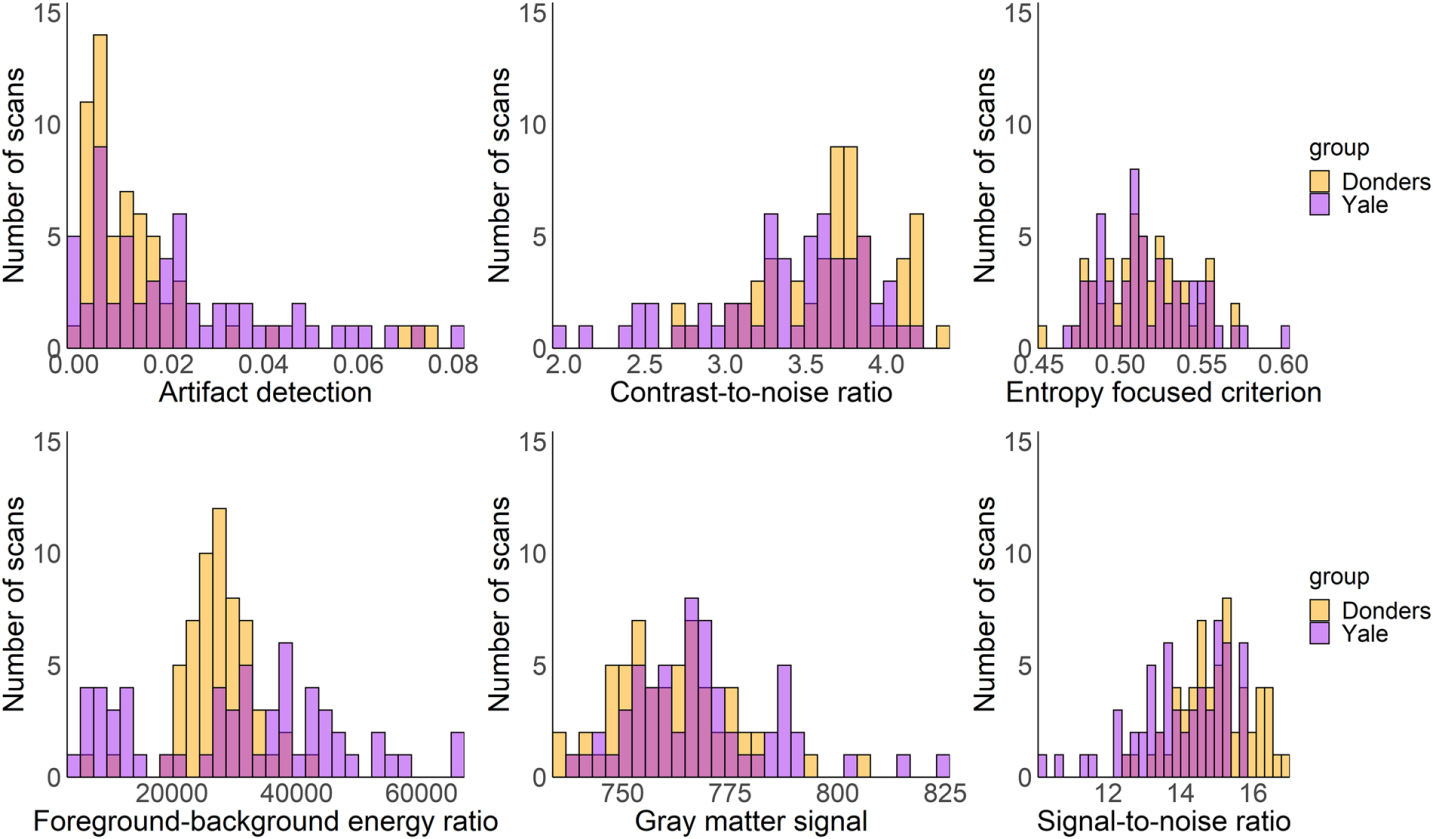
Distribution of QC measures of T1-weighted data.

We used MRIQC (version 16.0) to obtain data quality measures in the shared data sample. We present the following metrics to assess anatomical MRI data quality (for more details on the MRI quality control metrics see Esteban *et al*.^37^): artifact detection measure (qi_1), which represents the percentage of voxels affected by motion, blurring, or ghosting artifacts^40^; the contrast-to-noise ratio (cnr)^41^, which indicates the contrast between white and gray matter; the entropy-focused criterion (efc)^42,43^, a proxy for motion, blurring, or ghosting; the foreground-background energy ratio (fber)^44^, which measures the difference in variances of voxels inside and outside the brain; the gray matter signal (summary_gm_mean), which reflects the average signal of gray matter; and the signal-to-noise ratio (snr_total), a measure of signal quality relative to background noise (Fig. 4; values summarized in Table 3). Those, and other output measures from MRIQC can be found in Supplementary Table 2.

**Table 3:** Summary statistics of QC measures of T1-weighted data.

T1 QC measure	Median	SD	Range
Artifact detection (qi_1)	0.01	0.02	0.00–0.08
Contrast-to-noise ratio (cnr)	3.62	0.47	1.95–4.33
Entropy focused criterion (efc)	0.51	0.03	0.45–0.60
Foreground-background energy ratio (fber)	28498.15	12191.10	3767.72–65322.84
Gray matter signal (summary_gm_mean)	763.64	15.65	734.63–823.85
Signal-to-noise ratio (snr_total)	14.63	1.25	10.12–16.84

### Functional scans

**Fig. 5:**
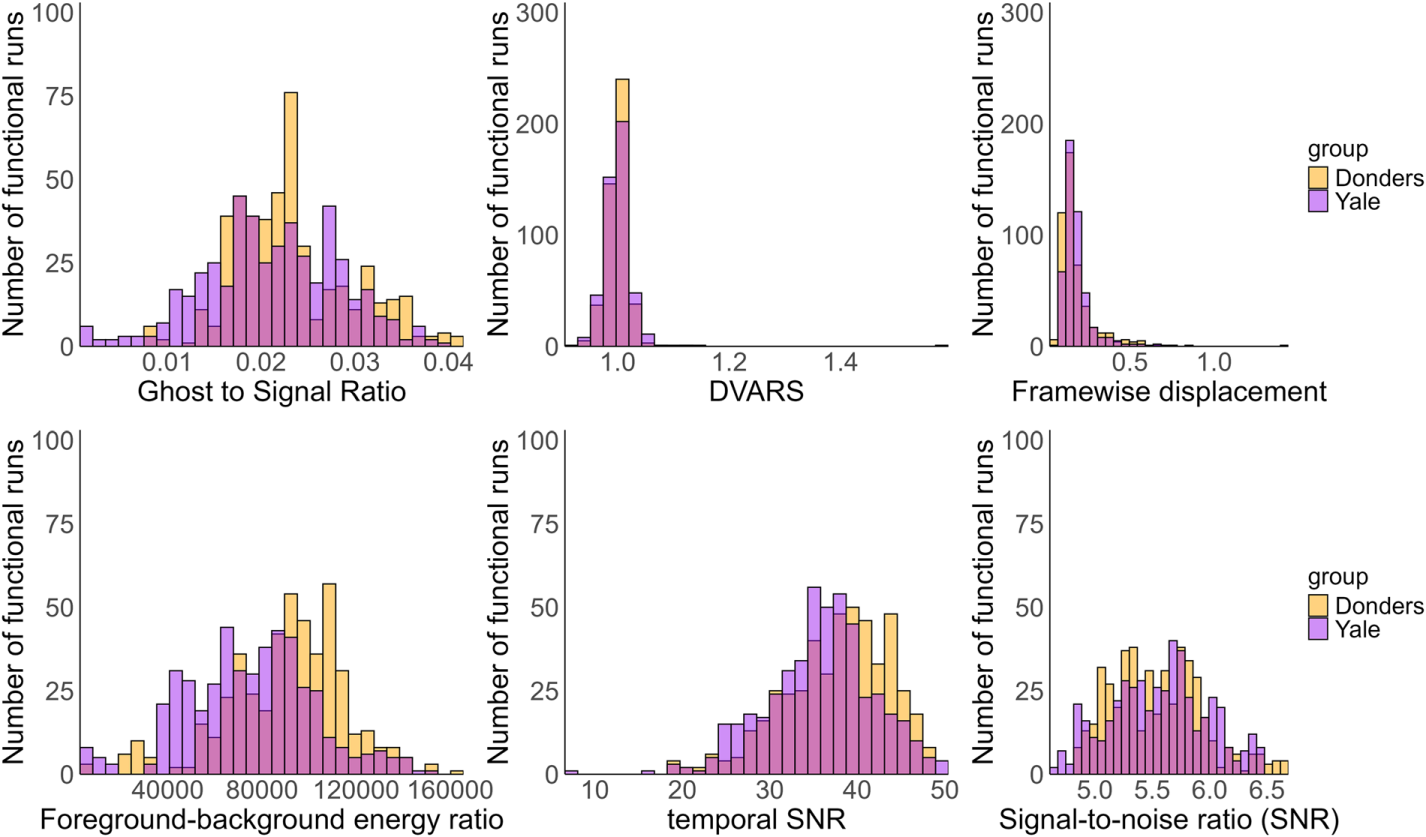
Distribution of QC measures of BOLD functional scans.

We evaluated the shared functional MRI data using the following quality metrics: ghost-to-signal ratio (gsr_y), which measures the ratio of signal intensity in areas affected by ghosting artifacts relative to inside the brain^45^; DVARS (dvars_vstd), which assesses how the voxel-wise signal changes from volume to volume at each timepoint; framewise displacement (fd_mean), a measure of average motion throughout the scan in millimeters; foreground-background energy ratio (fber), the average energy value within the head compared to regions outside the head^44^; temporal SNR (tsnr), the median of the signal-to-noise ratio across timepoints; and signal-to-noise ratio (snr), a relative measure of mean signal intensity relative to background noise (Fig. 5, values summarized in Table 4). These, along with other outputs from the MRIQC quality control can be found in Supplementary Table 3.

**Table 4:** Summary statistics of QC measures of BOLD functional scans.

Bold QC measures	Median	SD	Range
Ghost-to-signal ratio (gsr_y)	0.02	0.01	0.00–0.04
DVARS (dvars_vstd)	1.00	0.03	0.92–1.59
Framewise displacement (fd_mean)	0.15	0.11	0.06–1.44
Foreground-background energy ratio (fber)	85525	27431	3658–161107
Temporal SNR (tsnr)	37.31	6.15	8.00–50.38
Signal-to-noise ratio (snr)	5.56	0.41	4.67–6.68

## Usage Notes

The Terms of Use under which these data are made available can be found here. Data can be accessed via an active XNAT database (https://cogitate-data.ae.mpg.de/), which provides a user-friendly web interface for exploring and selectively downloading specific datasets. An API is available for programmatic data retrieval (https://wiki.xnat.org/documentation/the-xnat-api). Alternatively, comprehensive data bundles can be downloaded in a zip format from our website (https://www.arc-cogitate.com/data-release). Access to these resources requires users to create an account prior to downloading. Further details and instructions are available on our documentation page (https://cogitate-consortium.github.io/cogitate-data/).

The shared functional magnetic resonance imaging (fMRI) dataset was collected while participants engaged in the task outlined in the experimental design section. These data can be broadly used by researchers within consciousness neuroscience to ask questions related to theories of consciousness, but also beyond this field, for example to investigate various aspects of task-relevance, in visual neuroscience, time perception or sensitivity to stimulus categories.

### Data curation overview

Data curation procedures were performed to ensure compliance with privacy standards and maximize data usability. Project-level resources underwent scrutiny to remove unnecessary notifications, retaining critical metadata and standardized identifiers following the Brain Imaging Data Structure (BIDS).

Subject-specific documentation was excluded to protect participant confidentiality. Experiment-specific resources such as behavioral (BEH), case report forms (CRF), eye-tracking (ET), questionnaires (EXQU), and task triggers (LPT TRIGGER) were curated for standardization. Behavioral and eye-tracking data were anonymized by stripping personal identifiers and dates from the log files. CRF and questionnaire data originally in XLSX, DOCX and other proprietary formats were converted to JSON format to enhance data accessibility and interoperability.

For imaging data, a robust de-identification protocol was applied. DICOM files were anonymized comprehensively using automated scripts alongside XNAT tools, ensuring that headers and anatomical scans were free of identifiable information. Anatomical scans were specifically defaced by XNAT using the mask face tool^46^. Non-essential localizer and miscellaneous files were reviewed for potential identifying risks, selectively retained only after confirming the absence of identifiable information regarding facial or dental structures. Original DICOM files (DICOM_ORIG), snapshots, and additional extraneous data were removed, resulting in a clean, standardized, and fully anonymized fMRI dataset.

## Supplementary information

Supplementary Information

Supplementary Table 1: Trial count

Supplementary Table 2: T1 mriqc

Supplementary Table 3: Bold mriqc

## Data Availability

The data can be accessed through the following links: • Data Terms of Use: PDF file • Raw^47^ and BIDS^48^ fMRI Data (Bundle Format): Data Bundles • Raw and BIDS fMRI Data (XNAT^49^): XNAT Portal

## Code availability

The codebase is implemented in Python and MATLAB. For an overview of the code and environment setup instructions, please refer to the README file in the repository. The code can be accessed through the following links:

• Experiment Code^50^: GitHub - Experiment Code

• Preprocessing and Analysis Code^51^: GitHub - Analysis Code

For more information, please visit the COGITATE Data wiki, and the COGITATE website.

**Sample data and code demo.** Sample data from four participants (two per testing site) can be found here. We provide bids converted data (./bids/) as well as preprocessed data (./bids/derivatives/fmriprep/ and ./bids/derivatives/freesurfer/). We also provide data quality measures that can be found in ./bids/derivatives/mriqc/. At the link, instructions can be found to try out the analyses using these data.
