# Supplementary material for: An open-access multi-site fMRI dataset for investigating conscious visual perception
Source: Sci Data. 2026 May 7;13:779. doi: 10.1038/s41597-026-07377-y (PMC13213011; doi:10.1038/s41597-026-07377-y)
Supplement: Supplementary file 1 — Supplementary Information [file 41597_2026_7377_MOESM1_ESM.pdf]

COGITATE DATA RELEASE

fMRI Standard Operating Procedure (SOP)

Version	Author(s)
1.0	Khalaf, A.
**Date**	**Editor(s)**
December 19, 2023	Brown, T.

TABLE OF CONTENTS

1. fMRI Technical Checklist 1
1.1 Before subject arrives 1
1.2 Participant setup 1
1.3 Equipment Setup: Experiment PC and Connections 2
1.4 Equipment Setup: Eye Tracking 2
1.5 Experiment Code Setup: 3
1.5 Experiment Procedure 3
1.6 Scanning sequences & notes: Experiment 1 4
1.7 Scanning sequences & notes: Experiment 2 4
1.8 End of the Experiment 5
1.9 Data Saving and Sharing 5

1. fMRI Technical Checklist

Subject ID: _____________ Session: ________ Date: ____________

1.1 Before subject arrives

1.1.1. Have the following forms ready:

- MRI safety sheet
- COVID questionnaire
- COVID information sheet
- signed consent form
- exit questionnaire
- case report form (CRF)

1.1.2 Get the experiment laptop, the charger, and the eye chart.

1.2 Participant setup

- *Experiment 1:* Bring the participant to the lab and check their visual acuity at 20-feet distance from Snellen chart and note down the last row they can read.

- Take the participant to one of the MRRC behavioral rooms.
- Ask the participant to complete the MRI safety sheet, COVID questionnaire, and demographics form.
- Ask the participant to take a pregnancy test in case the participant is female
- *Experiment 1:* Explain the task instructions
- *Experiment 2:* Explain task instructions briefly and have the participant complete the preparation module
- Ask the participant whether he/she needs to use the restroom
- De-metal the participant
- Ask the participant to walk through the full-body metal detector
- Take the participant to the MR control room and hand the informed consent, MRI safety questionnaire, and the PI billing number to the MR technician to review.
- Siemens scanners have an 'Additional information' field. Ask the technician to set it to:
    - Experiment 1: Set it to S?1xx_V1
    - Experiment 2: Set it to S?1xx_V2
- The MR technician will take the subject to the magnet and instruct the participant on placing earbuds.
- Ask the technician to use the optoacoustic headphones
- *Experiment 1:* Ask the technician to place subject’s right index finger on the blue button
- *Experiment 2:* Ask the technician to place subject’s right index finger on the blue button and the left index finger on the red button

1.3 Equipment Setup: Experiment PC and Connections

- Plug in the experiment laptop to a power source
- Double check display refresh rate (60 Hz)
- Double check display resolution (1920×1080)
- Make sure there is no abnormality on stimulus displays
- Connect the button box and the projector to the experiment laptop via USB
- *Experiment 2:* Connect the audio jack to the experiment laptop
- Connect the experiment laptop via ethernet to the EyeLink 1000 Plus then turn on the EyeLink computer and initiate the software
- Open a text editor and ask the subject to press right index then middle index finger buttons. You should see the number 12.
- Move the cursor around the edges of the screen and see if the subject still sees it.

1.4 Equipment Setup: Eye Tracking

- Adjust eye tracking camera focus

- Optimize corneal reflection and pupil thresholds
- Switch off the lights.

1.5 Experiment Code Setup:

1.5.1 Experiment 1

- Open MATLAB 2019b and switch directory to runExp1.m directory
- Run: runExp1(participant_ID) [note IDs are: 1XX for real acquisition]

1.5.2 Experiment 2

- Run ‘Orange & Blue - A Tale of Falling Essences.exe
- Check if screen resolution = 1920x1080; graphic quality = ultra; Eyelink must be in camera mode.
- Enter participant ID [note IDs are: 1XX for real acquisition]
- Use Run ID = B for practice outside scanner.
- Use Run ID = 1 for full MRI session inside scanner (increment to 2, 3, … for restarts)

1.5 Experiment Procedure

- Scan localizer while starting the task.
- When the instruction screen pops up, tell the subject that we will scan during the practice, which starts after the instructions, and that the scans will continue sometime after the practice is over
- The subject reads instructions, practice levels starts, and we start the T1
- Practice finished; the T1 continues; we immediately run the head scout and the 1st inverted scan
- Explain eye tracking instructions to the subject
- Confirm the camera lens is optimally focused
- Optimize corneal reflection and pupil thresholds if needed
- Proceed with calibration and validation.
- Ask if the subject is ready to start
- Start run 1
- *Experiment 1:* Manually stop MR sequence a few seconds after performance screen is shown, but before clicking space bar to continue to next run. Click space to continue the ‘we will continue shortly screen’ only when the scanner is NOT scanning.
- *Experiment 2*: Manually stop MR sequence when leaderboard is shown.
- Proceed normally and communicate with the participant between runs to make sure that they understand the task and they take breaks as much as they need.

- Give the tech the account number and ask them to give you the study pa number
- *Experiment 2*: After it ends, run EVC localizer
- Open matlab 2019b. Switch directory to run EVC_localizer.m
- Enter participant ID note IDs are: 1XX for real acquisition and run number (usually 1, unless restarted localizer)

1.6 Scanning sequences & notes: Experiment 1

- Localizer
- T1: MPRAGE (during practice; continue with headscout + DC1 immediately)
- Headscout
- DistCor1: Inv. ET calibration (explain calibration)
- R1: MB4
- R2: MB4
- R3: MB4
- R4: MB4
- DistCor2: Inv. (run during ‘we will continue shortly’ screen)
- R5: MB4
- R6: MB4
- R7: MB4
- R8: MB4
- DistCor3: Inv.

1.7 Scanning sequences & notes: Experiment 2

- Localizer
- HeadscoutET calibration
- DistCor1: Inv. (during practice)
- VG1: MB4
- VG2: MB4
- VG3: MB4
- VG4: MB4
- DistCor2: Inv.
- VG5: MB4
- VG6: MB4
- VG7: MB4
- VG8: MB4
- DistCor3: Inv.
- Replay1: MB4
- Replay2: MB4
- Replay3: MB4

- Replay4: MB4
- DistCor4: Inv.
- EVCLoc: MB4

1.8 End of the Experiment

- Ask the participant to fill the exit questionnaire
- Give the participant his allowance, and make him sign the receipt

1.9 Data Saving and Sharing

Before leaving the control room, make sure that the following data types are saved:

- Eyetracker data
- Behavioral data
- PsychToolBox Code

Note: At Yale, the MRI technician uploads the structural and functional MRI data to the MRI center (MRRC) server. The data will be available for access in 1-2 hours post session. If the session is performed at night, the data might not be available until the next day.

- Get the MRI data from the MRRC server when available

- Before upload to XNAT:
    - Run anonymization script on ET data

- Upload data to XNAT: MRI data, ET data, Behavioural log files, Codes, Case report form, Questionnaire.

Table of contents

\\USER
DavRic
SiteProtocols
Blumenfeld_Templeton_Ex1
localizer_32
Anat_t1_mprage_sag_ipat2_1p0iso
AAHead_Scout
DC1_cmrr_2iso_mb4_TR1500_inv
DurR1_cmrr_2iso_mb4_TR1500
DurR2_cmrr_2iso_mb4_TR1500
DurR3_cmrr_2iso_mb4_TR1500
DurR4_cmrr_2iso_mb4_TR1500
DC2_cmrr_2iso_mb4_TR1500_inv
DurR5_cmrr_2iso_mb4_TR1500
DurR6_cmrr_2iso_mb4_TR1500
DurR7_cmrr_2iso_mb4_TR1500
DurR8_cmrr_2iso_mb4_TR1500
DC3_cmrr_2iso_mb4_TR1500_inv

**\\USER\DavRic\SiteProtocols\Blumenfeld_Templeton_Ex1\localizer_32**
TA: 0:12 PM: REF Voxel size: 0.5×0.5×7.0 mmPAT: Off Rel. SNR: 1.00 : fl

Properties

Prio recon	On
Load images to viewer	On
Inline movie	Off
Auto store images	On
Load images to stamp segments	On
Load images to graphic segments	On
Auto open inline display	Off
Auto close inline display	Off
Start measurement without further preparation	On
Wait for user to start	Off
Start measurements	Single measurement

Routine

Slice group	1
Slices	1
Dist. factor	20 %
Position	L0.0 A45.0 H0.0 mm
Orientation	Sagittal
Phase enc. dir.	A >> P
Slice group	2
Slices	1
Dist. factor	20 %
Position	L0.0 A20.0 H0.0 mm
Orientation	Transversal
Phase enc. dir.	A >> P
Slice group	3
Slices	1
Dist. factor	20 %
Position	L0.0 A20.0 H0.0 mm
Orientation	Coronal
Phase enc. dir.	R >> L
AutoAlign	---
Phase oversampling	0 %
FoV read	250 mm
FoV phase	100.0 %
Slice thickness	7.0 mm
TR	7.5 ms
TE	3.69 ms
Averages	2
Concatenations	3
Filter	Prescan Normalize, Elliptical filter
Coil elements	HC1-7;NC1,2

Contrast - Common

TR	7.5 ms
TE	3.69 ms
TD	0 ms
MTC	Off
Magn. preparation	None
Flip angle	20 deg
Fat suppr.	None
Water suppr.	None
SWI	Off

Contrast - Dynamic

Averages	2
Averaging mode	Short term
Reconstruction	Magnitude
Measurements	1

Contrast - Dynamic

Multiple series	Each measurement

Resolution - Common

FoV read	250 mm
FoV phase	100.0 %
Slice thickness	7.0 mm
Base resolution	256
Phase resolution	91 %
Phase partial Fourier	Off
Interpolation	On

Resolution - iPAT

PAT mode	None

Resolution - Filter Image

Image Filter	Off
Distortion Corr.	Off
Prescan Normalize	On
Unfiltered images	Off
Normalize	Off
B1 filter	Off

Resolution - Filter Rawdata

Raw filter	Off
Elliptical filter	On

Geometry - Common

Slice group	1
Slices	1
Dist. factor	20 %
Position	L0.0 A45.0 H0.0 mm
Orientation	Sagittal
Phase enc. dir.	A >> P
Slice group	2
Slices	1
Dist. factor	20 %
Position	L0.0 A20.0 H0.0 mm
Orientation	Transversal
Phase enc. dir.	A >> P
Slice group	3
Slices	1
Dist. factor	20 %
Position	L0.0 A20.0 H0.0 mm
Orientation	Coronal
Phase enc. dir.	R >> L
FoV read	250 mm
FoV phase	100.0 %
Slice thickness	7.0 mm
TR	7.5 ms
Multi-slice mode	Sequential
Series	Interleaved
Concatenations	3

Geometry - AutoAlign

Slice group	1
Position	L0.0 A45.0 H0.0 mm
Orientation	Sagittal
Phase enc. dir.	A >> P
Slice group	2
Position	L0.0 A20.0 H0.0 mm

Geometry - AutoAlign

Orientation	Transversal
Phase enc. dir.	A >> P
Slice group	3
Position	L0.0 A20.0 H0.0 mm
Orientation	Coronal
Phase enc. dir.	R >> L
AutoAlign	---
Initial Position	L0.0 A45.0 H0.0
L	0.0 mm
A	45.0 mm
H	0.0 mm
Initial Rotation	0.00 deg
Initial Orientation	Sagittal

Geometry - Saturation

Saturation mode	Standard
Fat suppr.	None
Water suppr.	None
Special sat.	None

System - Miscellaneous

Positioning mode	REF
Table position	H
Table position	0 mm
MSMA	S - C - T
Sagittal	R >> L
Coronal	A >> P
Transversal	F >> H
Coil Combine Mode	Sum of Squares
Save uncombined	Off
Matrix Optimization	Off
AutoAlign	---
Coil Select Mode	Off - All

System - Adjustments

B0 Shim mode	Tune up
B1 Shim mode	TrueForm
Adjust with body coil	Off
Confirm freq. adjustment	Off
Assume Dominant Fat	Off
Assume Silicone	Off
Adjustment Tolerance	Auto

System - Adjust Volume

Position	Isocenter
Orientation	Transversal
Rotation	0.00 deg
A >> P	263 mm
R >> L	350 mm
F >> H	350 mm
Reset	Off

System - pTx Volumes

B1 Shim mode	TrueForm
Excitation	Slice-sel.

System - Tx/Rx

Frequency 1H	123.253461 MHz
Correction factor	1
Gain	High
Img. Scale Cor.	1.000
Reset	Off
? Ref. amplitude 1H	0.000 V

Physio - Signal1

1st Signal/Mode	None
TR	7.5 ms
Concatenations	3
Segments	1

Physio - Cardiac

Magn. preparation	None
Fat suppr.	None
Dark blood	Off
FoV read	250 mm
FoV phase	100.0 %
Phase resolution	91 %

Physio - PACE

Resp. control	Off
Concatenations	3

Inline - Common

Subtract	Off
Measurements	1
StdDev	Off
Liver registration	Off
Save original images	On

Inline - MIP

MIP-Sag	Off
MIP-Cor	Off
MIP-Tra	Off
MIP-Time	Off
Save original images	On

Inline - Soft Tissue

Wash - In	Off
Wash - Out	Off
TTP	Off
PEI	Off
MIP - time	Off
Measurements	1

Inline - Composing

Distortion Corr.	Off

Sequence - Part 1

Introduction	On
Dimension	2D
Phase stabilisation	Off
Asymmetric echo	Allowed
Contrasts	1
Flow comp.	No
Multi-slice mode	Sequential
Bandwidth	320 Hz/Px

Sequence - Part 2

Segments	1
Acoustic noise reduction	None
RF pulse type	Fast
Gradient mode	Fast
Excitation	Slice-sel.
RF spoiling	On

Sequence - Assistant

Mode	Off
Allowed delay	0 s

\\USER\DavRic\SiteProtocols\Blumenfeld_Templeton_Ex1\Anat_t1_mprage_sag_ipat2_1p0iso
TA: 5:21 PM: REF Voxel size: 1.0×1.0×1.0 mmPAT: 2 Rel. SNR: 1.00 : tfl

Properties

Prio recon	Off
Load images to viewer	On
Inline movie	Off
Auto store images	On
Load images to stamp segments	Off
Load images to graphic segments	Off
Auto open inline display	Off
Auto close inline display	Off
Start measurement without further preparation	Off
Wait for user to start	On
Start measurements	Single measurement

Routine

Slab group	1
Slabs	1
Dist. factor	50 %
Position	Isocenter
Orientation	Sagittal
Phase enc. dir.	A >> P
AutoAlign	Head > Brain
Phase oversampling	0 %
Slice oversampling	0.0 %
Slices per slab	192
FoV read	256 mm
FoV phase	100.0 %
Slice thickness	1.00 mm
TR	2300.0 ms
TE	3.03 ms
Averages	1
Concatenations	1
Filter	Prescan Normalize, Elliptical filter
Coil elements	HEA;HEP

Contrast - Common

TR	2300.0 ms
TE	3.03 ms
Magn. preparation	Non-sel. IR
TI	1100 ms
Flip angle	8 deg
Fat suppr.	None
Water suppr.	None

Contrast - Dynamic

Averages	1
Averaging mode	Long term
Reconstruction	Magnitude
Measurements	1
Multiple series	Each measurement

Resolution - Common

FoV read	256 mm
FoV phase	100.0 %
Slice thickness	1.00 mm
Base resolution	256
Phase resolution	100 %
Slice resolution	100 %
Phase partial Fourier	Off
Slice partial Fourier	Off

Resolution - Common

Interpolation	Off

Resolution - iPAT

PAT mode	GRAPPA
Accel. factor PE	2
Ref. lines PE	24
Accel. factor 3D	1
Reference scan mode	Integrated

Resolution - Filter Image

Image Filter	Off
Distortion Corr.	Off
Prescan Normalize	On
Unfiltered images	Off
Normalize	Off
B1 filter	Off

Resolution - Filter Rawdata

Raw filter	Off
Elliptical filter	On

Geometry - Common

Slab group	1
Slabs	1
Dist. factor	50 %
Position	Isocenter
Orientation	Sagittal
Phase enc. dir.	A >> P
Slice oversampling	0.0 %
Slices per slab	192
FoV read	256 mm
FoV phase	100.0 %
Slice thickness	1.00 mm
TR	2300.0 ms
Multi-slice mode	Single shot
Series	Ascending
Concatenations	1

Geometry - AutoAlign

Slab group	1
Position	Isocenter
Orientation	Sagittal
Phase enc. dir.	A >> P
AutoAlign	Head > Brain
Initial Position	Isocenter
L	0.0 mm
P	0.0 mm
H	0.0 mm
Initial Rotation	0.00 deg
Initial Orientation	Sagittal

Geometry - Navigator

System - Miscellaneous

Positioning mode	REF
Table position	H
Table position	0 mm
MSMA	S - C - T
Sagittal	R >> L
Coronal	A >> P

System - Miscellaneous

Transversal	F >> H
Coil Combine Mode	Adaptive Combine
Save uncombined	Off
Matrix Optimization	Off
AutoAlign	Head > Brain
Coil Select Mode	Default

System - Adjustments

B0 Shim mode	Standard
B1 Shim mode	TrueForm
Adjust with body coil	On
Confirm freq. adjustment	Off
Assume Dominant Fat	Off
Assume Silicone	Off
Adjustment Tolerance	Auto

System - Adjust Volume

Position	Isocenter
Orientation	Sagittal
Rotation	0.00 deg
A >> P	256 mm
F >> H	256 mm
R >> L	192 mm
Reset	Off

System - pTx Volumes

B1 Shim mode	TrueForm
Excitation	Non-sel.

System - Tx/Rx

Frequency 1H	123.253461 MHz
Correction factor	1
Gain	Low
Img. Scale Cor.	1.000
Reset	Off
? Ref. amplitude 1H	0.000 V

Physio - Signal1

1st Signal/Mode	None
TR	2300.0 ms
Concatenations	1

Physio - Cardiac

Magn. preparation	Non-sel. IR
TI	1100 ms
Fat suppr.	None
Dark blood	Off
FoV read	256 mm
FoV phase	100.0 %
Phase resolution	100 %

Physio - PACE

Resp. control	Off
Concatenations	1

Inline - Common

Subtract	Off
Measurements	1
StdDev	Off
Save original images	On

Inline - MIP

MIP-Sag	Off

Inline - MIP

MIP-Cor	Off
MIP-Tra	Off
MIP-Time	Off
Save original images	On

Inline - Composing

Distortion Corr.	Off

Sequence - Part 1

Introduction	On
Dimension	3D
Elliptical scanning	Off
Reordering	Linear
Asymmetric echo	Allowed
Flow comp.	No
Multi-slice mode	Single shot
Echo spacing	8.9 ms
Bandwidth	130 Hz/Px

Sequence - Part 2

RF pulse type	Normal
Gradient mode	Normal
Excitation	Non-sel.
RF spoiling	On
Incr. Gradient spoiling	Off
Turbo factor	192

Sequence - Assistant

Mode	Off

\\USER\DavRic\SiteProtocols\Blumenfeld_Templeton_Ex1\AAHead_Scout

TA: 0:14 PM: REF Voxel size: 1.6×1.6×1.6 mmPAT: 3 Rel. SNR: 1.00 : fl

Properties

Prio recon	Off
Load images to viewer	On
Inline movie	Off
Auto store images	On
Load images to stamp segments	Off
Load images to graphic segments	On
Auto open inline display	Off
Auto close inline display	Off
Start measurement without further preparation	On
Wait for user to start	Off
Start measurements	Single measurement

Routine

Slab group	1
Slabs	1
Dist. factor	20 %
Position	L0.0 A45.0 H0.0 mm
Orientation	Sagittal
Phase enc. dir.	A >> P
Phase oversampling	0 %
Slice oversampling	0.0 %
Slices per slab	128
FoV read	260 mm
FoV phase	100.0 %
Slice thickness	1.6 mm
TR	3.15 ms
TE	1.37 ms
Averages	1
Concatenations	1
Filter	Prescan Normalize
Coil elements	HE1-4

Contrast - Common

TR	3.15 ms
TE	1.37 ms
Flip angle	8 deg

Contrast - Dynamic

Averages	1
Averaging mode	Short term
Reconstruction	Magnitude
Measurements	1

Resolution - Common

FoV read	260 mm
FoV phase	100.0 %
Slice thickness	1.6 mm
Base resolution	160
Phase resolution	100 %
Slice resolution	69 %
Phase partial Fourier	6/8
Slice partial Fourier	6/8
Trajectory	Cartesian

Resolution - iPAT

PAT mode	GRAPPA
Accel. factor PE	3
Ref. lines PE	24
Accel. factor 3D	1

Resolution - iPAT

Reference scan mode	Integrated

Resolution - Filter Image

Image Filter	Off
Distortion Corr.	Off
Prescan Normalize	On
Unfiltered images	Off
Normalize	Off
B1 filter	Off

Resolution - Filter Rawdata

Raw filter	Off
Elliptical filter	Off

Geometry - Common

Slab group	1
Slabs	1
Dist. factor	20 %
Position	L0.0 A45.0 H0.0 mm
Orientation	Sagittal
Phase enc. dir.	A >> P
Slice oversampling	0.0 %
Slices per slab	128
FoV read	260 mm
FoV phase	100.0 %
Slice thickness	1.6 mm
TR	3.15 ms
Multi-slice mode	Sequential
Series	Ascending
Concatenations	1

Geometry - AutoAlign

Slab group	1
Position	L0.0 A45.0 H0.0 mm
Orientation	Sagittal
Phase enc. dir.	A >> P
Initial Position	Isocenter
L	0.0 mm
P	0.0 mm
H	0.0 mm
Initial Rotation	0.00 deg
Initial Orientation	Transversal

System - Miscellaneous

Positioning mode	REF
Table position	H
Table position	0 mm
MSMA	S - C - T
Sagittal	R >> L
Coronal	A >> P
Transversal	F >> H
Coil Combine Mode	Adaptive Combine
Save uncombined	Off
Matrix Optimization	Off
Coil Select Mode	Off - AutoCoilSelect

System - Adjustments

B0 Shim mode	Tune up
B1 Shim mode	TrueForm
Adjust with body coil	Off

System - Adjustments

Confirm freq. adjustment	Off
Assume Dominant Fat	Off
Assume Silicone	Off
Adjustment Tolerance	Auto

System - Adjust Volume

Position	Isocenter
Orientation	Transversal
Rotation	0.00 deg
A >> P	263 mm
R >> L	350 mm
F >> H	350 mm
Reset	Off

System - pTx Volumes

B1 Shim mode	TrueForm
Excitation	Non-sel.

System - Tx/Rx

Frequency 1H	123.253461 MHz
Correction factor	1
Gain	Low
Img. Scale Cor.	1.000
Reset	Off
? Ref. amplitude 1H	0.000 V

Physio - PACE

Resp. control	Off
Concatenations	1

Inline - Common

Flip angle	8 deg
Measurements	1
Time to center	6.2 s

Inline - Inline

Subtract	Off
Measurements	1
StdDev	Off
Save original images	On

Inline - MIP

MIP-Sag	Off
MIP-Cor	Off
MIP-Tra	Off
MIP-Time	Off
Save original images	On

Inline - Composing

Distortion Corr.	Off

Sequence - Part 1

Introduction	On
Dimension	3D
Asymmetric echo	Weak
Contrasts	1
Multi-slice mode	Sequential
Bandwidth	540 Hz/Px

Sequence - Part 2

RF pulse type	Fast
Gradient mode	Normal
Excitation	Non-sel.

Sequence - Part 2

RF spoiling	On

Sequence - Assistant

Mode	Off

\\USER\DavRic\SiteProtocols\Blumenfeld_Templeton_Ex1\DC1_cmrr_2iso_mb4_TR1500_inv
TA: 0:18 PM: REF Voxel size: 2.0×2.0×2.0 mmPAT: Off Rel. SNR: 1.00 : epfid

Properties

Prio recon	Off
Load images to viewer	On
Inline movie	Off
Auto store images	On
Load images to stamp segments	Off
Load images to graphic segments	Off
Auto open inline display	Off
Auto close inline display	Off
Start measurement without further preparation	Off
Wait for user to start	On
Start measurements	Single measurement

Routine

Slice group	1
Slices	68
Dist. factor	0 %
Position	L0.0 P3.0 H6.0 mm
Orientation	T > C-20.0
Phase enc. dir.	A >> P
AutoAlign	Head > Brain
Phase oversampling	0 %
FoV read	210 mm
FoV phase	100.0 %
Slice thickness	2.00 mm
TR	1500 ms
TE	39.60 ms
Multi-band accel. factor	4
Filter	Prescan Normalize
Coil elements	HC4,6,7

Contrast - Common

TR	1500 ms
TE	39.60 ms
MTC	Off
Magn. preparation	None
Flip angle	75 deg
Fat suppr.	Fat sat.

Contrast - Dynamic

Averaging mode	Long term
Reconstruction	Magnitude
Measurements	5
Delay in TR	0 ms
Multiple series	Off

Resolution - Common

FoV read	210 mm
FoV phase	100.0 %
Slice thickness	2.00 mm
Base resolution	104
Phase resolution	100 %
Phase partial Fourier	Off
Interpolation	Off

Resolution - iPAT

PAT mode	None

Resolution - Filter Image

Distortion Corr.	Off

Resolution - Filter Image

Prescan Normalize	On

Resolution - Filter Rawdata

Raw filter	Off
Elliptical filter	Off
Hamming	Off

Geometry - Common

Slice group	1
Slices	68
Dist. factor	0 %
Position	L0.0 P3.0 H6.0 mm
Orientation	T > C-20.0
Phase enc. dir.	A >> P
FoV read	210 mm
FoV phase	100.0 %
Slice thickness	2.00 mm
TR	1500 ms
Multi-slice mode	Interleaved
Series	Interleaved
Multi-band accel. factor	4

Geometry - AutoAlign

Slice group	1
Position	L0.0 P3.0 H6.0 mm
Orientation	T > C-20.0
Phase enc. dir.	A >> P
AutoAlign	Head > Brain
Initial Position	L0.0 P3.0 H6.0
L	0.0 mm
P	3.0 mm
H	6.0 mm
Initial Rotation	0.00 deg
Initial Orientation	T > C
T > C	-20.0
> S	0.0

Geometry - Saturation

Fat suppr.	Fat sat.
Special sat.	None

System - Miscellaneous

Positioning mode	REF
Table position	H
Table position	0 mm
MSMA	S - C - T
Sagittal	R >> L
Coronal	A >> P
Transversal	F >> H
Coil Combine Mode	Sum of Squares
Matrix Optimization	Off
AutoAlign	Head > Brain
Coil Select Mode	Default

System - Adjustments

B0 Shim mode	Brain
B1 Shim mode	TrueForm
Adjust with body coil	Off
Confirm freq. adjustment	Off
Assume Dominant Fat	Off

System - Adjustments

Assume Silicone	Off
Adjustment Tolerance	Auto

System - Adjust Volume

Position	L0.0 P3.0 H6.0 mm
Orientation	T > C-20.0
Rotation	0.00 deg
A >> P	210 mm
R >> L	210 mm
F >> H	136 mm
Reset	Off

System - pTx Volumes

B1 Shim mode	TrueForm
Excitation	Standard

System - Tx/Rx

Frequency 1H	123.253461 MHz
Correction factor	1
Gain	High
Img. Scale Cor.	1.000
Reset	Off
? Ref. amplitude 1H	0.000 V

Physio - Signal1

1st Signal/Mode	None
TR	1500 ms
Multi-band accel. factor	4

BOLD

GLM Statistics	Off
Dynamic t-maps	Off
Ignore meas. at start	0
Ignore after transition	0
Model transition states	On
Temp. highpass filter	On
Threshold	4.00
Paradigm size	20
Meas[1]	Baseline
Meas[2]	Baseline
Meas[3]	Active
Meas[4]	Active
Meas[5]	Active
Meas[6]	Active
Meas[7]	Active
Meas[8]	Active
Meas[9]	Active
Meas[10]	Active
Meas[11]	Active
Meas[12]	Active
Meas[13]	Active
Meas[14]	Active
Meas[15]	Active
Meas[16]	Active
Meas[17]	Active
Meas[18]	Active
Meas[19]	Active
Meas[20]	Active
Motion correction	Off
Spatial filter	Off
Measurements	5
Delay in TR	0 ms
Multiple series	Off

Sequence - Part 1

Introduction	Off
Contrasts	1
Flow comp.	No
Multi-slice mode	Interleaved
Free echo spacing	Off
Echo spacing	0.58 ms
Bandwidth	2090 Hz/Px

Sequence - Part 2

EPI factor	104
Gradient mode	Performance
Excitation	Standard
RF spoiling	Off

Sequence - Special

Excite pulse duration	6000 us
Single-band images	On
MB LeakBlock kernel	On
MB dual kernel	Off
MB RF phase scramble	On
SENSE1 coil combine	On
Invert RO/PE polarity	On
Disable freq. update	Off
Force equal slice timing	Off
Online multi-band recon.	Online
FFT scale factor	1.00
Physio recording	Off
Triggering scheme	Standard

\\USER\DavRic\SiteProtocols\Blumenfeld_Templeton_Ex1\DurR1_cmrr_2iso_mb4_TR1500

TA: 12:41 PM: REF Voxel size: 2.0×2.0×2.0 mmPAT: Off Rel. SNR: 1.00 : epfid

Properties

Prio recon	Off
Load images to viewer	On
Inline movie	Off
Auto store images	On
Load images to stamp segments	Off
Load images to graphic segments	Off
Auto open inline display	Off
Auto close inline display	Off
Start measurement without further preparation	Off
Wait for user to start	On
Start measurements	Single measurement

Routine

Slice group	1
Slices	68
Dist. factor	0 %
Position	L0.0 P3.0 H6.0 mm
Orientation	T > C-20.0
Phase enc. dir.	A >> P
AutoAlign	Head > Brain
Phase oversampling	0 %
FoV read	210 mm
FoV phase	100.0 %
Slice thickness	2.00 mm
TR	1500 ms
TE	39.60 ms
Multi-band accel. factor	4
Filter	Prescan Normalize
Coil elements	HC4,6,7

Contrast - Common

TR	1500 ms
TE	39.60 ms
MTC	Off
Magn. preparation	None
Flip angle	75 deg
Fat suppr.	Fat sat.

Contrast - Dynamic

Averaging mode	Long term
Reconstruction	Magnitude
Measurements	500
Delay in TR	0 ms
Multiple series	Off

Resolution - Common

FoV read	210 mm
FoV phase	100.0 %
Slice thickness	2.00 mm
Base resolution	104
Phase resolution	100 %
Phase partial Fourier	Off
Interpolation	Off

Resolution - iPAT

PAT mode	None

Resolution - Filter Image

Distortion Corr.	Off

Resolution - Filter Image

Prescan Normalize	On

Resolution - Filter Rawdata

Raw filter	Off
Elliptical filter	Off
Hamming	Off

Geometry - Common

Slice group	1
Slices	68
Dist. factor	0 %
Position	L0.0 P3.0 H6.0 mm
Orientation	T > C-20.0
Phase enc. dir.	A >> P
FoV read	210 mm
FoV phase	100.0 %
Slice thickness	2.00 mm
TR	1500 ms
Multi-slice mode	Interleaved
Series	Interleaved
Multi-band accel. factor	4

Geometry - AutoAlign

Slice group	1
Position	L0.0 P3.0 H6.0 mm
Orientation	T > C-20.0
Phase enc. dir.	A >> P
AutoAlign	Head > Brain
Initial Position	L0.0 P3.0 H6.0
L	0.0 mm
P	3.0 mm
H	6.0 mm
Initial Rotation	0.00 deg
Initial Orientation	T > C
T > C	-20.0
> S	0.0

Geometry - Saturation

Fat suppr.	Fat sat.
Special sat.	None

System - Miscellaneous

Positioning mode	REF
Table position	H
Table position	0 mm
MSMA	S - C - T
Sagittal	R >> L
Coronal	A >> P
Transversal	F >> H
Coil Combine Mode	Sum of Squares
Matrix Optimization	Off
AutoAlign	Head > Brain
Coil Select Mode	Default

System - Adjustments

B0 Shim mode	Brain
B1 Shim mode	TrueForm
Adjust with body coil	Off
Confirm freq. adjustment	Off
Assume Dominant Fat	Off

System - Adjustments

Assume Silicone	Off
Adjustment Tolerance	Auto

System - Adjust Volume

Position	L0.0 P3.0 H6.0 mm
Orientation	T > C-20.0
Rotation	0.00 deg
A >> P	210 mm
R >> L	210 mm
F >> H	136 mm
Reset	Off

System - pTx Volumes

B1 Shim mode	TrueForm
Excitation	Standard

System - Tx/Rx

Frequency 1H	123.253461 MHz
Correction factor	1
Gain	High
Img. Scale Cor.	1.000
Reset	Off
? Ref. amplitude 1H	0.000 V

Physio - Signal1

1st Signal/Mode	None
TR	1500 ms
Multi-band accel. factor	4

BOLD

GLM Statistics	Off
Dynamic t-maps	Off
Ignore meas. at start	0
Ignore after transition	0
Model transition states	On
Temp. highpass filter	On
Threshold	4.00
Paradigm size	20
Meas[1]	Baseline
Meas[2]	Baseline
Meas[3]	Active
Meas[4]	Active
Meas[5]	Active
Meas[6]	Active
Meas[7]	Active
Meas[8]	Active
Meas[9]	Active
Meas[10]	Active
Meas[11]	Active
Meas[12]	Active
Meas[13]	Active
Meas[14]	Active
Meas[15]	Active
Meas[16]	Active
Meas[17]	Active
Meas[18]	Active
Meas[19]	Active
Meas[20]	Active
Motion correction	Off
Spatial filter	Off
Measurements	500
Delay in TR	0 ms
Multiple series	Off

Sequence - Part 1

Introduction	Off
Contrasts	1
Flow comp.	No
Multi-slice mode	Interleaved
Free echo spacing	Off
Echo spacing	0.58 ms
Bandwidth	2090 Hz/Px

Sequence - Part 2

EPI factor	104
Gradient mode	Performance
Excitation	Standard
RF spoiling	Off

Sequence - Special

Excite pulse duration	6000 us
Single-band images	On
MB LeakBlock kernel	On
MB dual kernel	Off
MB RF phase scramble	On
SENSE1 coil combine	On
Invert RO/PE polarity	Off
Disable freq. update	Off
Force equal slice timing	Off
Online multi-band recon.	Online
FFT scale factor	1.00
Physio recording	Off
Triggering scheme	Standard

\\USER\DavRic\SiteProtocols\Blumenfeld_Templeton_Ex1\DurR2_cmrr_2iso_mb4_TR1500
TA: 12:41 PM: REF Voxel size: 2.0×2.0×2.0 mmPAT: Off Rel. SNR: 1.00 : epfid

Properties

Prio recon	Off
Load images to viewer	On
Inline movie	Off
Auto store images	On
Load images to stamp segments	Off
Load images to graphic segments	Off
Auto open inline display	Off
Auto close inline display	Off
Start measurement without further preparation	Off
Wait for user to start	On
Start measurements	Single measurement

Routine

Slice group	1
Slices	68
Dist. factor	0 %
Position	L0.0 P3.0 H6.0 mm
Orientation	T > C-20.0
Phase enc. dir.	A >> P
AutoAlign	Head > Brain
Phase oversampling	0 %
FoV read	210 mm
FoV phase	100.0 %
Slice thickness	2.00 mm
TR	1500 ms
TE	39.60 ms
Multi-band accel. factor	4
Filter	Prescan Normalize
Coil elements	HC4,6,7

Contrast - Common

TR	1500 ms
TE	39.60 ms
MTC	Off
Magn. preparation	None
Flip angle	75 deg
Fat suppr.	Fat sat.

Contrast - Dynamic

Averaging mode	Long term
Reconstruction	Magnitude
Measurements	500
Delay in TR	0 ms
Multiple series	Off

Resolution - Common

FoV read	210 mm
FoV phase	100.0 %
Slice thickness	2.00 mm
Base resolution	104
Phase resolution	100 %
Phase partial Fourier	Off
Interpolation	Off

Resolution - iPAT

PAT mode	None

Resolution - Filter Image

Distortion Corr.	Off

Resolution - Filter Image

Prescan Normalize	On

Resolution - Filter Rawdata

Raw filter	Off
Elliptical filter	Off
Hamming	Off

Geometry - Common

Slice group	1
Slices	68
Dist. factor	0 %
Position	L0.0 P3.0 H6.0 mm
Orientation	T > C-20.0
Phase enc. dir.	A >> P
FoV read	210 mm
FoV phase	100.0 %
Slice thickness	2.00 mm
TR	1500 ms
Multi-slice mode	Interleaved
Series	Interleaved
Multi-band accel. factor	4

Geometry - AutoAlign

Slice group	1
Position	L0.0 P3.0 H6.0 mm
Orientation	T > C-20.0
Phase enc. dir.	A >> P
AutoAlign	Head > Brain
Initial Position	L0.0 P3.0 H6.0
L	0.0 mm
P	3.0 mm
H	6.0 mm
Initial Rotation	0.00 deg
Initial Orientation	T > C
T > C	-20.0
> S	0.0

Geometry - Saturation

Fat suppr.	Fat sat.
Special sat.	None

System - Miscellaneous

Positioning mode	REF
Table position	H
Table position	0 mm
MSMA	S - C - T
Sagittal	R >> L
Coronal	A >> P
Transversal	F >> H
Coil Combine Mode	Sum of Squares
Matrix Optimization	Off
AutoAlign	Head > Brain
Coil Select Mode	Default

System - Adjustments

B0 Shim mode	Brain
B1 Shim mode	TrueForm
Adjust with body coil	Off
Confirm freq. adjustment	Off
Assume Dominant Fat	Off

System - Adjustments

Assume Silicone	Off
Adjustment Tolerance	Auto

System - Adjust Volume

Position	L0.0 P3.0 H6.0 mm
Orientation	T > C-20.0
Rotation	0.00 deg
A >> P	210 mm
R >> L	210 mm
F >> H	136 mm
Reset	Off

System - pTx Volumes

B1 Shim mode	TrueForm
Excitation	Standard

System - Tx/Rx

Frequency 1H	123.253461 MHz
Correction factor	1
Gain	High
Img. Scale Cor.	1.000
Reset	Off
? Ref. amplitude 1H	0.000 V

Physio - Signal1

1st Signal/Mode	None
TR	1500 ms
Multi-band accel. factor	4

BOLD

GLM Statistics	Off
Dynamic t-maps	Off
Ignore meas. at start	0
Ignore after transition	0
Model transition states	On
Temp. highpass filter	On
Threshold	4.00
Paradigm size	20
Meas[1]	Baseline
Meas[2]	Baseline
Meas[3]	Active
Meas[4]	Active
Meas[5]	Active
Meas[6]	Active
Meas[7]	Active
Meas[8]	Active
Meas[9]	Active
Meas[10]	Active
Meas[11]	Active
Meas[12]	Active
Meas[13]	Active
Meas[14]	Active
Meas[15]	Active
Meas[16]	Active
Meas[17]	Active
Meas[18]	Active
Meas[19]	Active
Meas[20]	Active
Motion correction	Off
Spatial filter	Off
Measurements	500
Delay in TR	0 ms
Multiple series	Off

Sequence - Part 1

Introduction	Off
Contrasts	1
Flow comp.	No
Multi-slice mode	Interleaved
Free echo spacing	Off
Echo spacing	0.58 ms
Bandwidth	2090 Hz/Px

Sequence - Part 2

EPI factor	104
Gradient mode	Performance
Excitation	Standard
RF spoiling	Off

Sequence - Special

Excite pulse duration	6000 us
Single-band images	On
MB LeakBlock kernel	On
MB dual kernel	Off
MB RF phase scramble	On
SENSE1 coil combine	On
Invert RO/PE polarity	Off
Disable freq. update	Off
Force equal slice timing	Off
Online multi-band recon.	Online
FFT scale factor	1.00
Physio recording	Off
Triggering scheme	Standard

\\USER\DavRic\SiteProtocols\Blumenfeld_Templeton_Ex1\DurR3_cmrr_2iso_mb4_TR1500

TA: 12:41 PM: REF Voxel size: 2.0×2.0×2.0 mmPAT: Off Rel. SNR: 1.00 : epfid

Properties

Prio recon	Off
Load images to viewer	On
Inline movie	Off
Auto store images	On
Load images to stamp segments	Off
Load images to graphic segments	Off
Auto open inline display	Off
Auto close inline display	Off
Start measurement without further preparation	Off
Wait for user to start	On
Start measurements	Single measurement

Routine

Slice group	1
Slices	68
Dist. factor	0 %
Position	L0.0 P3.0 H6.0 mm
Orientation	T > C-20.0
Phase enc. dir.	A >> P
AutoAlign	Head > Brain
Phase oversampling	0 %
FoV read	210 mm
FoV phase	100.0 %
Slice thickness	2.00 mm
TR	1500 ms
TE	39.60 ms
Multi-band accel. factor	4
Filter	Prescan Normalize
Coil elements	HC4,6,7

Contrast - Common

TR	1500 ms
TE	39.60 ms
MTC	Off
Magn. preparation	None
Flip angle	75 deg
Fat suppr.	Fat sat.

Contrast - Dynamic

Averaging mode	Long term
Reconstruction	Magnitude
Measurements	500
Delay in TR	0 ms
Multiple series	Off

Resolution - Common

FoV read	210 mm
FoV phase	100.0 %
Slice thickness	2.00 mm
Base resolution	104
Phase resolution	100 %
Phase partial Fourier	Off
Interpolation	Off

Resolution - iPAT

PAT mode	None

Resolution - Filter Image

Distortion Corr.	Off

Resolution - Filter Image

Prescan Normalize	On

Resolution - Filter Rawdata

Raw filter	Off
Elliptical filter	Off
Hamming	Off

Geometry - Common

Slice group	1
Slices	68
Dist. factor	0 %
Position	L0.0 P3.0 H6.0 mm
Orientation	T > C-20.0
Phase enc. dir.	A >> P
FoV read	210 mm
FoV phase	100.0 %
Slice thickness	2.00 mm
TR	1500 ms
Multi-slice mode	Interleaved
Series	Interleaved
Multi-band accel. factor	4

Geometry - AutoAlign

Slice group	1
Position	L0.0 P3.0 H6.0 mm
Orientation	T > C-20.0
Phase enc. dir.	A >> P
AutoAlign	Head > Brain
Initial Position	L0.0 P3.0 H6.0
L	0.0 mm
P	3.0 mm
H	6.0 mm
Initial Rotation	0.00 deg
Initial Orientation	T > C
T > C	-20.0
> S	0.0

Geometry - Saturation

Fat suppr.	Fat sat.
Special sat.	None

System - Miscellaneous

Positioning mode	REF
Table position	H
Table position	0 mm
MSMA	S - C - T
Sagittal	R >> L
Coronal	A >> P
Transversal	F >> H
Coil Combine Mode	Sum of Squares
Matrix Optimization	Off
AutoAlign	Head > Brain
Coil Select Mode	Default

System - Adjustments

B0 Shim mode	Brain
B1 Shim mode	TrueForm
Adjust with body coil	Off
Confirm freq. adjustment	Off
Assume Dominant Fat	Off

System - Adjustments

Assume Silicone	Off
Adjustment Tolerance	Auto

System - Adjust Volume

Position	L0.0 P3.0 H6.0 mm
Orientation	T > C-20.0
Rotation	0.00 deg
A >> P	210 mm
R >> L	210 mm
F >> H	136 mm
Reset	Off

System - pTx Volumes

B1 Shim mode	TrueForm
Excitation	Standard

System - Tx/Rx

Frequency 1H	123.253461 MHz
Correction factor	1
Gain	High
Img. Scale Cor.	1.000
Reset	Off
? Ref. amplitude 1H	0.000 V

Physio - Signal1

1st Signal/Mode	None
TR	1500 ms
Multi-band accel. factor	4

BOLD

GLM Statistics	Off
Dynamic t-maps	Off
Ignore meas. at start	0
Ignore after transition	0
Model transition states	On
Temp. highpass filter	On
Threshold	4.00
Paradigm size	20
Meas[1]	Baseline
Meas[2]	Baseline
Meas[3]	Active
Meas[4]	Active
Meas[5]	Active
Meas[6]	Active
Meas[7]	Active
Meas[8]	Active
Meas[9]	Active
Meas[10]	Active
Meas[11]	Active
Meas[12]	Active
Meas[13]	Active
Meas[14]	Active
Meas[15]	Active
Meas[16]	Active
Meas[17]	Active
Meas[18]	Active
Meas[19]	Active
Meas[20]	Active
Motion correction	Off
Spatial filter	Off
Measurements	500
Delay in TR	0 ms
Multiple series	Off

Sequence - Part 1

Introduction	Off
Contrasts	1
Flow comp.	No
Multi-slice mode	Interleaved
Free echo spacing	Off
Echo spacing	0.58 ms
Bandwidth	2090 Hz/Px

Sequence - Part 2

EPI factor	104
Gradient mode	Performance
Excitation	Standard
RF spoiling	Off

Sequence - Special

Excite pulse duration	6000 us
Single-band images	On
MB LeakBlock kernel	On
MB dual kernel	Off
MB RF phase scramble	On
SENSE1 coil combine	On
Invert RO/PE polarity	Off
Disable freq. update	Off
Force equal slice timing	Off
Online multi-band recon.	Online
FFT scale factor	1.00
Physio recording	Off
Triggering scheme	Standard

\\USER\DavRic\SiteProtocols\Blumenfeld_Templeton_Ex1\DurR4_cmrr_2iso_mb4_TR1500
TA: 12:41 PM: REF Voxel size: 2.0×2.0×2.0 mmPAT: Off Rel. SNR: 1.00 : epfid

Properties

Prio recon	Off
Load images to viewer	On
Inline movie	Off
Auto store images	On
Load images to stamp segments	Off
Load images to graphic segments	Off
Auto open inline display	Off
Auto close inline display	Off
Start measurement without further preparation	Off
Wait for user to start	On
Start measurements	Single measurement

Routine

Slice group	1
Slices	68
Dist. factor	0 %
Position	L0.0 P3.0 H6.0 mm
Orientation	T > C-20.0
Phase enc. dir.	A >> P
AutoAlign	Head > Brain
Phase oversampling	0 %
FoV read	210 mm
FoV phase	100.0 %
Slice thickness	2.00 mm
TR	1500 ms
TE	39.60 ms
Multi-band accel. factor	4
Filter	Prescan Normalize
Coil elements	HC4,6,7

Contrast - Common

TR	1500 ms
TE	39.60 ms
MTC	Off
Magn. preparation	None
Flip angle	75 deg
Fat suppr.	Fat sat.

Contrast - Dynamic

Averaging mode	Long term
Reconstruction	Magnitude
Measurements	500
Delay in TR	0 ms
Multiple series	Off

Resolution - Common

FoV read	210 mm
FoV phase	100.0 %
Slice thickness	2.00 mm
Base resolution	104
Phase resolution	100 %
Phase partial Fourier	Off
Interpolation	Off

Resolution - iPAT

PAT mode	None

Resolution - Filter Image

Distortion Corr.	Off

Resolution - Filter Image

Prescan Normalize	On

Resolution - Filter Rawdata

Raw filter	Off
Elliptical filter	Off
Hamming	Off

Geometry - Common

Slice group	1
Slices	68
Dist. factor	0 %
Position	L0.0 P3.0 H6.0 mm
Orientation	T > C-20.0
Phase enc. dir.	A >> P
FoV read	210 mm
FoV phase	100.0 %
Slice thickness	2.00 mm
TR	1500 ms
Multi-slice mode	Interleaved
Series	Interleaved
Multi-band accel. factor	4

Geometry - AutoAlign

Slice group	1
Position	L0.0 P3.0 H6.0 mm
Orientation	T > C-20.0
Phase enc. dir.	A >> P
AutoAlign	Head > Brain
Initial Position	L0.0 P3.0 H6.0
L	0.0 mm
P	3.0 mm
H	6.0 mm
Initial Rotation	0.00 deg
Initial Orientation	T > C
T > C	-20.0
> S	0.0

Geometry - Saturation

Fat suppr.	Fat sat.
Special sat.	None

System - Miscellaneous

Positioning mode	REF
Table position	H
Table position	0 mm
MSMA	S - C - T
Sagittal	R >> L
Coronal	A >> P
Transversal	F >> H
Coil Combine Mode	Sum of Squares
Matrix Optimization	Off
AutoAlign	Head > Brain
Coil Select Mode	Default

System - Adjustments

B0 Shim mode	Brain
B1 Shim mode	TrueForm
Adjust with body coil	Off
Confirm freq. adjustment	Off
Assume Dominant Fat	Off

System - Adjustments

Assume Silicone	Off
Adjustment Tolerance	Auto

System - Adjust Volume

Position	L0.0 P3.0 H6.0 mm
Orientation	T > C-20.0
Rotation	0.00 deg
A >> P	210 mm
R >> L	210 mm
F >> H	136 mm
Reset	Off

System - pTx Volumes

B1 Shim mode	TrueForm
Excitation	Standard

System - Tx/Rx

Frequency 1H	123.253461 MHz
Correction factor	1
Gain	High
Img. Scale Cor.	1.000
Reset	Off
? Ref. amplitude 1H	0.000 V

Physio - Signal1

1st Signal/Mode	None
TR	1500 ms
Multi-band accel. factor	4

BOLD

GLM Statistics	Off
Dynamic t-maps	Off
Ignore meas. at start	0
Ignore after transition	0
Model transition states	On
Temp. highpass filter	On
Threshold	4.00
Paradigm size	20
Meas[1]	Baseline
Meas[2]	Baseline
Meas[3]	Active
Meas[4]	Active
Meas[5]	Active
Meas[6]	Active
Meas[7]	Active
Meas[8]	Active
Meas[9]	Active
Meas[10]	Active
Meas[11]	Active
Meas[12]	Active
Meas[13]	Active
Meas[14]	Active
Meas[15]	Active
Meas[16]	Active
Meas[17]	Active
Meas[18]	Active
Meas[19]	Active
Meas[20]	Active
Motion correction	Off
Spatial filter	Off
Measurements	500
Delay in TR	0 ms
Multiple series	Off

Sequence - Part 1

Introduction	Off
Contrasts	1
Flow comp.	No
Multi-slice mode	Interleaved
Free echo spacing	Off
Echo spacing	0.58 ms
Bandwidth	2090 Hz/Px

Sequence - Part 2

EPI factor	104
Gradient mode	Performance
Excitation	Standard
RF spoiling	Off

Sequence - Special

Excite pulse duration	6000 us
Single-band images	On
MB LeakBlock kernel	On
MB dual kernel	Off
MB RF phase scramble	On
SENSE1 coil combine	On
Invert RO/PE polarity	Off
Disable freq. update	Off
Force equal slice timing	Off
Online multi-band recon.	Online
FFT scale factor	1.00
Physio recording	Off
Triggering scheme	Standard

\\USER\DavRic\SiteProtocols\Blumenfeld_Templeton_Ex1\DC2_cmrr_2iso_mb4_TR1500_inv

TA: 0:18 PM: REF Voxel size: 2.0×2.0×2.0 mmPAT: Off Rel. SNR: 1.00 : epfid

Properties

Prio recon	Off
Load images to viewer	On
Inline movie	Off
Auto store images	On
Load images to stamp segments	Off
Load images to graphic segments	Off
Auto open inline display	Off
Auto close inline display	Off
Start measurement without further preparation	Off
Wait for user to start	On
Start measurements	Single measurement

Routine

Slice group	1
Slices	68
Dist. factor	0 %
Position	L0.0 P3.0 H6.0 mm
Orientation	T > C-20.0
Phase enc. dir.	A >> P
AutoAlign	Head > Brain
Phase oversampling	0 %
FoV read	210 mm
FoV phase	100.0 %
Slice thickness	2.00 mm
TR	1500 ms
TE	39.60 ms
Multi-band accel. factor	4
Filter	Prescan Normalize
Coil elements	HC4,6,7

Contrast - Common

TR	1500 ms
TE	39.60 ms
MTC	Off
Magn. preparation	None
Flip angle	75 deg
Fat suppr.	Fat sat.

Contrast - Dynamic

Averaging mode	Long term
Reconstruction	Magnitude
Measurements	5
Delay in TR	0 ms
Multiple series	Off

Resolution - Common

FoV read	210 mm
FoV phase	100.0 %
Slice thickness	2.00 mm
Base resolution	104
Phase resolution	100 %
Phase partial Fourier	Off
Interpolation	Off

Resolution - iPAT

PAT mode	None

Resolution - Filter Image

Distortion Corr.	Off

Resolution - Filter Image

Prescan Normalize	On

Resolution - Filter Rawdata

Raw filter	Off
Elliptical filter	Off
Hamming	Off

Geometry - Common

Slice group	1
Slices	68
Dist. factor	0 %
Position	L0.0 P3.0 H6.0 mm
Orientation	T > C-20.0
Phase enc. dir.	A >> P
FoV read	210 mm
FoV phase	100.0 %
Slice thickness	2.00 mm
TR	1500 ms
Multi-slice mode	Interleaved
Series	Interleaved
Multi-band accel. factor	4

Geometry - AutoAlign

Slice group	1
Position	L0.0 P3.0 H6.0 mm
Orientation	T > C-20.0
Phase enc. dir.	A >> P
AutoAlign	Head > Brain
Initial Position	L0.0 P3.0 H6.0
L	0.0 mm
P	3.0 mm
H	6.0 mm
Initial Rotation	0.00 deg
Initial Orientation	T > C
T > C	-20.0
> S	0.0

Geometry - Saturation

Fat suppr.	Fat sat.
Special sat.	None

System - Miscellaneous

Positioning mode	REF
Table position	H
Table position	0 mm
MSMA	S - C - T
Sagittal	R >> L
Coronal	A >> P
Transversal	F >> H
Coil Combine Mode	Sum of Squares
Matrix Optimization	Off
AutoAlign	Head > Brain
Coil Select Mode	Default

System - Adjustments

B0 Shim mode	Brain
B1 Shim mode	TrueForm
Adjust with body coil	Off
Confirm freq. adjustment	Off
Assume Dominant Fat	Off

System - Adjustments

Assume Silicone	Off
Adjustment Tolerance	Auto

System - Adjust Volume

Position	L0.0 P3.0 H6.0 mm
Orientation	T > C-20.0
Rotation	0.00 deg
A >> P	210 mm
R >> L	210 mm
F >> H	136 mm
Reset	Off

System - pTx Volumes

B1 Shim mode	TrueForm
Excitation	Standard

System - Tx/Rx

Frequency 1H	123.253461 MHz
Correction factor	1
Gain	High
Img. Scale Cor.	1.000
Reset	Off
? Ref. amplitude 1H	0.000 V

Physio - Signal1

1st Signal/Mode	None
TR	1500 ms
Multi-band accel. factor	4

BOLD

GLM Statistics	Off
Dynamic t-maps	Off
Ignore meas. at start	0
Ignore after transition	0
Model transition states	On
Temp. highpass filter	On
Threshold	4.00
Paradigm size	20
Meas[1]	Baseline
Meas[2]	Baseline
Meas[3]	Active
Meas[4]	Active
Meas[5]	Active
Meas[6]	Active
Meas[7]	Active
Meas[8]	Active
Meas[9]	Active
Meas[10]	Active
Meas[11]	Active
Meas[12]	Active
Meas[13]	Active
Meas[14]	Active
Meas[15]	Active
Meas[16]	Active
Meas[17]	Active
Meas[18]	Active
Meas[19]	Active
Meas[20]	Active
Motion correction	Off
Spatial filter	Off
Measurements	5
Delay in TR	0 ms
Multiple series	Off

Sequence - Part 1

Introduction	Off
Contrasts	1
Flow comp.	No
Multi-slice mode	Interleaved
Free echo spacing	Off
Echo spacing	0.58 ms
Bandwidth	2090 Hz/Px

Sequence - Part 2

EPI factor	104
Gradient mode	Performance
Excitation	Standard
RF spoiling	Off

Sequence - Special

Excite pulse duration	6000 us
Single-band images	On
MB LeakBlock kernel	On
MB dual kernel	Off
MB RF phase scramble	On
SENSE1 coil combine	On
Invert RO/PE polarity	On
Disable freq. update	Off
Force equal slice timing	Off
Online multi-band recon.	Online
FFT scale factor	1.00
Physio recording	Off
Triggering scheme	Standard

\\USER\DavRic\SiteProtocols\Blumenfeld_Templeton_Ex1\DurR5_cmrr_2iso_mb4_TR1500
TA: 12:41 PM: REF Voxel size: 2.0×2.0×2.0 mmPAT: Off Rel. SNR: 1.00 : epfid

Properties

Prio recon	Off
Load images to viewer	On
Inline movie	Off
Auto store images	On
Load images to stamp segments	Off
Load images to graphic segments	Off
Auto open inline display	Off
Auto close inline display	Off
Start measurement without further preparation	Off
Wait for user to start	On
Start measurements	Single measurement

Routine

Slice group	1
Slices	68
Dist. factor	0 %
Position	L0.0 P3.0 H6.0 mm
Orientation	T > C-20.0
Phase enc. dir.	A >> P
AutoAlign	Head > Brain
Phase oversampling	0 %
FoV read	210 mm
FoV phase	100.0 %
Slice thickness	2.00 mm
TR	1500 ms
TE	39.60 ms
Multi-band accel. factor	4
Filter	Prescan Normalize
Coil elements	HC4,6,7

Contrast - Common

TR	1500 ms
TE	39.60 ms
MTC	Off
Magn. preparation	None
Flip angle	75 deg
Fat suppr.	Fat sat.

Contrast - Dynamic

Averaging mode	Long term
Reconstruction	Magnitude
Measurements	500
Delay in TR	0 ms
Multiple series	Off

Resolution - Common

FoV read	210 mm
FoV phase	100.0 %
Slice thickness	2.00 mm
Base resolution	104
Phase resolution	100 %
Phase partial Fourier	Off
Interpolation	Off

Resolution - iPAT

PAT mode	None

Resolution - Filter Image

Distortion Corr.	Off

Resolution - Filter Image

Prescan Normalize	On

Resolution - Filter Rawdata

Raw filter	Off
Elliptical filter	Off
Hamming	Off

Geometry - Common

Slice group	1
Slices	68
Dist. factor	0 %
Position	L0.0 P3.0 H6.0 mm
Orientation	T > C-20.0
Phase enc. dir.	A >> P
FoV read	210 mm
FoV phase	100.0 %
Slice thickness	2.00 mm
TR	1500 ms
Multi-slice mode	Interleaved
Series	Interleaved
Multi-band accel. factor	4

Geometry - AutoAlign

Slice group	1
Position	L0.0 P3.0 H6.0 mm
Orientation	T > C-20.0
Phase enc. dir.	A >> P
AutoAlign	Head > Brain
Initial Position	L0.0 P3.0 H6.0
L	0.0 mm
P	3.0 mm
H	6.0 mm
Initial Rotation	0.00 deg
Initial Orientation	T > C
T > C	-20.0
> S	0.0

Geometry - Saturation

Fat suppr.	Fat sat.
Special sat.	None

System - Miscellaneous

Positioning mode	REF
Table position	H
Table position	0 mm
MSMA	S - C - T
Sagittal	R >> L
Coronal	A >> P
Transversal	F >> H
Coil Combine Mode	Sum of Squares
Matrix Optimization	Off
AutoAlign	Head > Brain
Coil Select Mode	Default

System - Adjustments

B0 Shim mode	Brain
B1 Shim mode	TrueForm
Adjust with body coil	Off
Confirm freq. adjustment	Off
Assume Dominant Fat	Off

System - Adjustments

Assume Silicone	Off
Adjustment Tolerance	Auto

System - Adjust Volume

Position	L0.0 P3.0 H6.0 mm
Orientation	T > C-20.0
Rotation	0.00 deg
A >> P	210 mm
R >> L	210 mm
F >> H	136 mm
Reset	Off

System - pTx Volumes

B1 Shim mode	TrueForm
Excitation	Standard

System - Tx/Rx

Frequency 1H	123.253461 MHz
Correction factor	1
Gain	High
Img. Scale Cor.	1.000
Reset	Off
? Ref. amplitude 1H	0.000 V

Physio - Signal1

1st Signal/Mode	None
TR	1500 ms
Multi-band accel. factor	4

BOLD

GLM Statistics	Off
Dynamic t-maps	Off
Ignore meas. at start	0
Ignore after transition	0
Model transition states	On
Temp. highpass filter	On
Threshold	4.00
Paradigm size	20
Meas[1]	Baseline
Meas[2]	Baseline
Meas[3]	Active
Meas[4]	Active
Meas[5]	Active
Meas[6]	Active
Meas[7]	Active
Meas[8]	Active
Meas[9]	Active
Meas[10]	Active
Meas[11]	Active
Meas[12]	Active
Meas[13]	Active
Meas[14]	Active
Meas[15]	Active
Meas[16]	Active
Meas[17]	Active
Meas[18]	Active
Meas[19]	Active
Meas[20]	Active
Motion correction	Off
Spatial filter	Off
Measurements	500
Delay in TR	0 ms
Multiple series	Off

Sequence - Part 1

Introduction	Off
Contrasts	1
Flow comp.	No
Multi-slice mode	Interleaved
Free echo spacing	Off
Echo spacing	0.58 ms
Bandwidth	2090 Hz/Px

Sequence - Part 2

EPI factor	104
Gradient mode	Performance
Excitation	Standard
RF spoiling	Off

Sequence - Special

Excite pulse duration	6000 us
Single-band images	On
MB LeakBlock kernel	On
MB dual kernel	Off
MB RF phase scramble	On
SENSE1 coil combine	On
Invert RO/PE polarity	Off
Disable freq. update	Off
Force equal slice timing	Off
Online multi-band recon.	Online
FFT scale factor	1.00
Physio recording	Off
Triggering scheme	Standard

\\USER\DavRic\SiteProtocols\Blumenfeld_Templeton_Ex1\DurR6_cmrr_2iso_mb4_TR1500

TA: 12:41 PM: REF Voxel size: 2.0×2.0×2.0 mmPAT: Off Rel. SNR: 1.00 : epfid

Properties

Prio recon	Off
Load images to viewer	On
Inline movie	Off
Auto store images	On
Load images to stamp segments	Off
Load images to graphic segments	Off
Auto open inline display	Off
Auto close inline display	Off
Start measurement without further preparation	Off
Wait for user to start	On
Start measurements	Single measurement

Routine

Slice group	1
Slices	68
Dist. factor	0 %
Position	L0.0 P3.0 H6.0 mm
Orientation	T > C-20.0
Phase enc. dir.	A >> P
AutoAlign	Head > Brain
Phase oversampling	0 %
FoV read	210 mm
FoV phase	100.0 %
Slice thickness	2.00 mm
TR	1500 ms
TE	39.60 ms
Multi-band accel. factor	4
Filter	Prescan Normalize
Coil elements	HC4,6,7

Contrast - Common

TR	1500 ms
TE	39.60 ms
MTC	Off
Magn. preparation	None
Flip angle	75 deg
Fat suppr.	Fat sat.

Contrast - Dynamic

Averaging mode	Long term
Reconstruction	Magnitude
Measurements	500
Delay in TR	0 ms
Multiple series	Off

Resolution - Common

FoV read	210 mm
FoV phase	100.0 %
Slice thickness	2.00 mm
Base resolution	104
Phase resolution	100 %
Phase partial Fourier	Off
Interpolation	Off

Resolution - iPAT

PAT mode	None

Resolution - Filter Image

Distortion Corr.	Off

Resolution - Filter Image

Prescan Normalize	On

Resolution - Filter Rawdata

Raw filter	Off
Elliptical filter	Off
Hamming	Off

Geometry - Common

Slice group	1
Slices	68
Dist. factor	0 %
Position	L0.0 P3.0 H6.0 mm
Orientation	T > C-20.0
Phase enc. dir.	A >> P
FoV read	210 mm
FoV phase	100.0 %
Slice thickness	2.00 mm
TR	1500 ms
Multi-slice mode	Interleaved
Series	Interleaved
Multi-band accel. factor	4

Geometry - AutoAlign

Slice group	1
Position	L0.0 P3.0 H6.0 mm
Orientation	T > C-20.0
Phase enc. dir.	A >> P
AutoAlign	Head > Brain
Initial Position	L0.0 P3.0 H6.0
L	0.0 mm
P	3.0 mm
H	6.0 mm
Initial Rotation	0.00 deg
Initial Orientation	T > C
T > C	-20.0
> S	0.0

Geometry - Saturation

Fat suppr.	Fat sat.
Special sat.	None

System - Miscellaneous

Positioning mode	REF
Table position	H
Table position	0 mm
MSMA	S - C - T
Sagittal	R >> L
Coronal	A >> P
Transversal	F >> H
Coil Combine Mode	Sum of Squares
Matrix Optimization	Off
AutoAlign	Head > Brain
Coil Select Mode	Default

System - Adjustments

B0 Shim mode	Brain
B1 Shim mode	TrueForm
Adjust with body coil	Off
Confirm freq. adjustment	Off
Assume Dominant Fat	Off

System - Adjustments

Assume Silicone	Off
Adjustment Tolerance	Auto

System - Adjust Volume

Position	L0.0 P3.0 H6.0 mm
Orientation	T > C-20.0
Rotation	0.00 deg
A >> P	210 mm
R >> L	210 mm
F >> H	136 mm
Reset	Off

System - pTx Volumes

B1 Shim mode	TrueForm
Excitation	Standard

System - Tx/Rx

Frequency 1H	123.253461 MHz
Correction factor	1
Gain	High
Img. Scale Cor.	1.000
Reset	Off
? Ref. amplitude 1H	0.000 V

Physio - Signal1

1st Signal/Mode	None
TR	1500 ms
Multi-band accel. factor	4

BOLD

GLM Statistics	Off
Dynamic t-maps	Off
Ignore meas. at start	0
Ignore after transition	0
Model transition states	On
Temp. highpass filter	On
Threshold	4.00
Paradigm size	20
Meas[1]	Baseline
Meas[2]	Baseline
Meas[3]	Active
Meas[4]	Active
Meas[5]	Active
Meas[6]	Active
Meas[7]	Active
Meas[8]	Active
Meas[9]	Active
Meas[10]	Active
Meas[11]	Active
Meas[12]	Active
Meas[13]	Active
Meas[14]	Active
Meas[15]	Active
Meas[16]	Active
Meas[17]	Active
Meas[18]	Active
Meas[19]	Active
Meas[20]	Active
Motion correction	Off
Spatial filter	Off
Measurements	500
Delay in TR	0 ms
Multiple series	Off

Sequence - Part 1

Introduction	Off
Contrasts	1
Flow comp.	No
Multi-slice mode	Interleaved
Free echo spacing	Off
Echo spacing	0.58 ms
Bandwidth	2090 Hz/Px

Sequence - Part 2

EPI factor	104
Gradient mode	Performance
Excitation	Standard
RF spoiling	Off

Sequence - Special

Excite pulse duration	6000 us
Single-band images	On
MB LeakBlock kernel	On
MB dual kernel	Off
MB RF phase scramble	On
SENSE1 coil combine	On
Invert RO/PE polarity	Off
Disable freq. update	Off
Force equal slice timing	Off
Online multi-band recon.	Online
FFT scale factor	1.00
Physio recording	Off
Triggering scheme	Standard

\\USER\DavRic\SiteProtocols\Blumenfeld_Templeton_Ex1\DurR7_cmrr_2iso_mb4_TR1500
TA: 12:41 PM: REF Voxel size: 2.0×2.0×2.0 mmPAT: Off Rel. SNR: 1.00 : epfid

Properties

Prio recon	Off
Load images to viewer	On
Inline movie	Off
Auto store images	On
Load images to stamp segments	Off
Load images to graphic segments	Off
Auto open inline display	Off
Auto close inline display	Off
Start measurement without further preparation	Off
Wait for user to start	On
Start measurements	Single measurement

Routine

Slice group	1
Slices	68
Dist. factor	0 %
Position	L0.0 P3.0 H6.0 mm
Orientation	T > C-20.0
Phase enc. dir.	A >> P
AutoAlign	Head > Brain
Phase oversampling	0 %
FoV read	210 mm
FoV phase	100.0 %
Slice thickness	2.00 mm
TR	1500 ms
TE	39.60 ms
Multi-band accel. factor	4
Filter	Prescan Normalize
Coil elements	HC4,6,7

Contrast - Common

TR	1500 ms
TE	39.60 ms
MTC	Off
Magn. preparation	None
Flip angle	75 deg
Fat suppr.	Fat sat.

Contrast - Dynamic

Averaging mode	Long term
Reconstruction	Magnitude
Measurements	500
Delay in TR	0 ms
Multiple series	Off

Resolution - Common

FoV read	210 mm
FoV phase	100.0 %
Slice thickness	2.00 mm
Base resolution	104
Phase resolution	100 %
Phase partial Fourier	Off
Interpolation	Off

Resolution - iPAT

PAT mode	None

Resolution - Filter Image

Distortion Corr.	Off

Resolution - Filter Image

Prescan Normalize	On

Resolution - Filter Rawdata

Raw filter	Off
Elliptical filter	Off
Hamming	Off

Geometry - Common

Slice group	1
Slices	68
Dist. factor	0 %
Position	L0.0 P3.0 H6.0 mm
Orientation	T > C-20.0
Phase enc. dir.	A >> P
FoV read	210 mm
FoV phase	100.0 %
Slice thickness	2.00 mm
TR	1500 ms
Multi-slice mode	Interleaved
Series	Interleaved
Multi-band accel. factor	4

Geometry - AutoAlign

Slice group	1
Position	L0.0 P3.0 H6.0 mm
Orientation	T > C-20.0
Phase enc. dir.	A >> P
AutoAlign	Head > Brain
Initial Position	L0.0 P3.0 H6.0
L	0.0 mm
P	3.0 mm
H	6.0 mm
Initial Rotation	0.00 deg
Initial Orientation	T > C
T > C	-20.0
> S	0.0

Geometry - Saturation

Fat suppr.	Fat sat.
Special sat.	None

System - Miscellaneous

Positioning mode	REF
Table position	H
Table position	0 mm
MSMA	S - C - T
Sagittal	R >> L
Coronal	A >> P
Transversal	F >> H
Coil Combine Mode	Sum of Squares
Matrix Optimization	Off
AutoAlign	Head > Brain
Coil Select Mode	Default

System - Adjustments

B0 Shim mode	Brain
B1 Shim mode	TrueForm
Adjust with body coil	Off
Confirm freq. adjustment	Off
Assume Dominant Fat	Off

System - Adjustments

Assume Silicone	Off
Adjustment Tolerance	Auto

System - Adjust Volume

Position	L0.0 P3.0 H6.0 mm
Orientation	T > C-20.0
Rotation	0.00 deg
A >> P	210 mm
R >> L	210 mm
F >> H	136 mm
Reset	Off

System - pTx Volumes

B1 Shim mode	TrueForm
Excitation	Standard

System - Tx/Rx

Frequency 1H	123.253461 MHz
Correction factor	1
Gain	High
Img. Scale Cor.	1.000
Reset	Off
? Ref. amplitude 1H	0.000 V

Physio - Signal1

1st Signal/Mode	None
TR	1500 ms
Multi-band accel. factor	4

BOLD

GLM Statistics	Off
Dynamic t-maps	Off
Ignore meas. at start	0
Ignore after transition	0
Model transition states	On
Temp. highpass filter	On
Threshold	4.00
Paradigm size	20
Meas[1]	Baseline
Meas[2]	Baseline
Meas[3]	Active
Meas[4]	Active
Meas[5]	Active
Meas[6]	Active
Meas[7]	Active
Meas[8]	Active
Meas[9]	Active
Meas[10]	Active
Meas[11]	Active
Meas[12]	Active
Meas[13]	Active
Meas[14]	Active
Meas[15]	Active
Meas[16]	Active
Meas[17]	Active
Meas[18]	Active
Meas[19]	Active
Meas[20]	Active
Motion correction	Off
Spatial filter	Off
Measurements	500
Delay in TR	0 ms
Multiple series	Off

Sequence - Part 1

Introduction	Off
Contrasts	1
Flow comp.	No
Multi-slice mode	Interleaved
Free echo spacing	Off
Echo spacing	0.58 ms
Bandwidth	2090 Hz/Px

Sequence - Part 2

EPI factor	104
Gradient mode	Performance
Excitation	Standard
RF spoiling	Off

Sequence - Special

Excite pulse duration	6000 us
Single-band images	On
MB LeakBlock kernel	On
MB dual kernel	Off
MB RF phase scramble	On
SENSE1 coil combine	On
Invert RO/PE polarity	Off
Disable freq. update	Off
Force equal slice timing	Off
Online multi-band recon.	Online
FFT scale factor	1.00
Physio recording	Off
Triggering scheme	Standard

\\USER\DavRic\SiteProtocols\Blumenfeld_Templeton_Ex1\DurR8_cmrr_2iso_mb4_TR1500
TA: 12:41 PM: REF Voxel size: 2.0×2.0×2.0 mmPAT: Off Rel. SNR: 1.00 : epfid

Properties

Prio recon	Off
Load images to viewer	On
Inline movie	Off
Auto store images	On
Load images to stamp segments	Off
Load images to graphic segments	Off
Auto open inline display	Off
Auto close inline display	Off
Start measurement without further preparation	Off
Wait for user to start	On
Start measurements	Single measurement

Routine

Slice group	1
Slices	68
Dist. factor	0 %
Position	L0.0 P3.0 H6.0 mm
Orientation	T > C-20.0
Phase enc. dir.	A >> P
AutoAlign	Head > Brain
Phase oversampling	0 %
FoV read	210 mm
FoV phase	100.0 %
Slice thickness	2.00 mm
TR	1500 ms
TE	39.60 ms
Multi-band accel. factor	4
Filter	Prescan Normalize
Coil elements	HC4,6,7

Contrast - Common

TR	1500 ms
TE	39.60 ms
MTC	Off
Magn. preparation	None
Flip angle	75 deg
Fat suppr.	Fat sat.

Contrast - Dynamic

Averaging mode	Long term
Reconstruction	Magnitude
Measurements	500
Delay in TR	0 ms
Multiple series	Off

Resolution - Common

FoV read	210 mm
FoV phase	100.0 %
Slice thickness	2.00 mm
Base resolution	104
Phase resolution	100 %
Phase partial Fourier	Off
Interpolation	Off

Resolution - iPAT

PAT mode	None

Resolution - Filter Image

Distortion Corr.	Off

Resolution - Filter Image

Prescan Normalize	On

Resolution - Filter Rawdata

Raw filter	Off
Elliptical filter	Off
Hamming	Off

Geometry - Common

Slice group	1
Slices	68
Dist. factor	0 %
Position	L0.0 P3.0 H6.0 mm
Orientation	T > C-20.0
Phase enc. dir.	A >> P
FoV read	210 mm
FoV phase	100.0 %
Slice thickness	2.00 mm
TR	1500 ms
Multi-slice mode	Interleaved
Series	Interleaved
Multi-band accel. factor	4

Geometry - AutoAlign

Slice group	1
Position	L0.0 P3.0 H6.0 mm
Orientation	T > C-20.0
Phase enc. dir.	A >> P
AutoAlign	Head > Brain
Initial Position	L0.0 P3.0 H6.0
L	0.0 mm
P	3.0 mm
H	6.0 mm
Initial Rotation	0.00 deg
Initial Orientation	T > C
T > C	-20.0
> S	0.0

Geometry - Saturation

Fat suppr.	Fat sat.
Special sat.	None

System - Miscellaneous

Positioning mode	REF
Table position	H
Table position	0 mm
MSMA	S - C - T
Sagittal	R >> L
Coronal	A >> P
Transversal	F >> H
Coil Combine Mode	Sum of Squares
Matrix Optimization	Off
AutoAlign	Head > Brain
Coil Select Mode	Default

System - Adjustments

B0 Shim mode	Brain
B1 Shim mode	TrueForm
Adjust with body coil	Off
Confirm freq. adjustment	Off
Assume Dominant Fat	Off

System - Adjustments

Assume Silicone	Off
Adjustment Tolerance	Auto

System - Adjust Volume

Position	L0.0 P3.0 H6.0 mm
Orientation	T > C-20.0
Rotation	0.00 deg
A >> P	210 mm
R >> L	210 mm
F >> H	136 mm
Reset	Off

System - pTx Volumes

B1 Shim mode	TrueForm
Excitation	Standard

System - Tx/Rx

Frequency 1H	123.253461 MHz
Correction factor	1
Gain	High
Img. Scale Cor.	1.000
Reset	Off
? Ref. amplitude 1H	0.000 V

Physio - Signal1

1st Signal/Mode	None
TR	1500 ms
Multi-band accel. factor	4

BOLD

GLM Statistics	Off
Dynamic t-maps	Off
Ignore meas. at start	0
Ignore after transition	0
Model transition states	On
Temp. highpass filter	On
Threshold	4.00
Paradigm size	20
Meas[1]	Baseline
Meas[2]	Baseline
Meas[3]	Active
Meas[4]	Active
Meas[5]	Active
Meas[6]	Active
Meas[7]	Active
Meas[8]	Active
Meas[9]	Active
Meas[10]	Active
Meas[11]	Active
Meas[12]	Active
Meas[13]	Active
Meas[14]	Active
Meas[15]	Active
Meas[16]	Active
Meas[17]	Active
Meas[18]	Active
Meas[19]	Active
Meas[20]	Active
Motion correction	Off
Spatial filter	Off
Measurements	500
Delay in TR	0 ms
Multiple series	Off

Sequence - Part 1

Introduction	Off
Contrasts	1
Flow comp.	No
Multi-slice mode	Interleaved
Free echo spacing	Off
Echo spacing	0.58 ms
Bandwidth	2090 Hz/Px

Sequence - Part 2

EPI factor	104
Gradient mode	Performance
Excitation	Standard
RF spoiling	Off

Sequence - Special

Excite pulse duration	6000 us
Single-band images	On
MB LeakBlock kernel	On
MB dual kernel	Off
MB RF phase scramble	On
SENSE1 coil combine	On
Invert RO/PE polarity	Off
Disable freq. update	Off
Force equal slice timing	Off
Online multi-band recon.	Online
FFT scale factor	1.00
Physio recording	Off
Triggering scheme	Standard

\\USER\DavRic\SiteProtocols\Blumenfeld_Templeton_Ex1\DC3_cmrr_2iso_mb4_TR1500_inv

TA: 0:18 PM: REF Voxel size: 2.0×2.0×2.0 mmPAT: Off Rel. SNR: 1.00 : epfid

Properties

Prio recon	Off
Load images to viewer	On
Inline movie	Off
Auto store images	On
Load images to stamp segments	Off
Load images to graphic segments	Off
Auto open inline display	Off
Auto close inline display	Off
Start measurement without further preparation	Off
Wait for user to start	On
Start measurements	Single measurement

Routine

Slice group	1
Slices	68
Dist. factor	0 %
Position	L0.0 P3.0 H6.0 mm
Orientation	T > C-20.0
Phase enc. dir.	A >> P
AutoAlign	Head > Brain
Phase oversampling	0 %
FoV read	210 mm
FoV phase	100.0 %
Slice thickness	2.00 mm
TR	1500 ms
TE	39.60 ms
Multi-band accel. factor	4
Filter	Prescan Normalize
Coil elements	HC4,6,7

Contrast - Common

TR	1500 ms
TE	39.60 ms
MTC	Off
Magn. preparation	None
Flip angle	75 deg
Fat suppr.	Fat sat.

Contrast - Dynamic

Averaging mode	Long term
Reconstruction	Magnitude
Measurements	5
Delay in TR	0 ms
Multiple series	Off

Resolution - Common

FoV read	210 mm
FoV phase	100.0 %
Slice thickness	2.00 mm
Base resolution	104
Phase resolution	100 %
Phase partial Fourier	Off
Interpolation	Off

Resolution - iPAT

PAT mode	None

Resolution - Filter Image

Distortion Corr.	Off

Resolution - Filter Image

Prescan Normalize	On

Resolution - Filter Rawdata

Raw filter	Off
Elliptical filter	Off
Hamming	Off

Geometry - Common

Slice group	1
Slices	68
Dist. factor	0 %
Position	L0.0 P3.0 H6.0 mm
Orientation	T > C-20.0
Phase enc. dir.	A >> P
FoV read	210 mm
FoV phase	100.0 %
Slice thickness	2.00 mm
TR	1500 ms
Multi-slice mode	Interleaved
Series	Interleaved
Multi-band accel. factor	4

Geometry - AutoAlign

Slice group	1
Position	L0.0 P3.0 H6.0 mm
Orientation	T > C-20.0
Phase enc. dir.	A >> P
AutoAlign	Head > Brain
Initial Position	L0.0 P3.0 H6.0
L	0.0 mm
P	3.0 mm
H	6.0 mm
Initial Rotation	0.00 deg
Initial Orientation	T > C
T > C	-20.0
> S	0.0

Geometry - Saturation

Fat suppr.	Fat sat.
Special sat.	None

System - Miscellaneous

Positioning mode	REF
Table position	H
Table position	0 mm
MSMA	S - C - T
Sagittal	R >> L
Coronal	A >> P
Transversal	F >> H
Coil Combine Mode	Sum of Squares
Matrix Optimization	Off
AutoAlign	Head > Brain
Coil Select Mode	Default

System - Adjustments

B0 Shim mode	Brain
B1 Shim mode	TrueForm
Adjust with body coil	Off
Confirm freq. adjustment	Off
Assume Dominant Fat	Off

System - Adjustments

Assume Silicone	Off
Adjustment Tolerance	Auto

System - Adjust Volume

Position	L0.0 P3.0 H6.0 mm
Orientation	T > C-20.0
Rotation	0.00 deg
A >> P	210 mm
R >> L	210 mm
F >> H	136 mm
Reset	Off

System - pTx Volumes

B1 Shim mode	TrueForm
Excitation	Standard

System - Tx/Rx

Frequency 1H	123.253461 MHz
Correction factor	1
Gain	High
Img. Scale Cor.	1.000
Reset	Off
? Ref. amplitude 1H	0.000 V

Physio - Signal1

1st Signal/Mode	None
TR	1500 ms
Multi-band accel. factor	4

BOLD

GLM Statistics	Off
Dynamic t-maps	Off
Ignore meas. at start	0
Ignore after transition	0
Model transition states	On
Temp. highpass filter	On
Threshold	4.00
Paradigm size	20
Meas[1]	Baseline
Meas[2]	Baseline
Meas[3]	Active
Meas[4]	Active
Meas[5]	Active
Meas[6]	Active
Meas[7]	Active
Meas[8]	Active
Meas[9]	Active
Meas[10]	Active
Meas[11]	Active
Meas[12]	Active
Meas[13]	Active
Meas[14]	Active
Meas[15]	Active
Meas[16]	Active
Meas[17]	Active
Meas[18]	Active
Meas[19]	Active
Meas[20]	Active
Motion correction	Off
Spatial filter	Off
Measurements	5
Delay in TR	0 ms
Multiple series	Off

Sequence - Part 1

Introduction	Off
Contrasts	1
Flow comp.	No
Multi-slice mode	Interleaved
Free echo spacing	Off
Echo spacing	0.58 ms
Bandwidth	2090 Hz/Px

Sequence - Part 2

EPI factor	104
Gradient mode	Performance
Excitation	Standard
RF spoiling	Off

Sequence - Special

Excite pulse duration	6000 us
Single-band images	On
MB LeakBlock kernel	On
MB dual kernel	Off
MB RF phase scramble	On
SENSE1 coil combine	On
Invert RO/PE polarity	On
Disable freq. update	Off
Force equal slice timing	Off
Online multi-band recon.	Online
FFT scale factor	1.00
Physio recording	Off
Triggering scheme	Standard
